# Reassessing endothelial-to-mesenchymal transition in mouse bone marrow: insights from lineage tracing models

**DOI:** 10.1038/s41467-023-44312-w

**Published:** 2023-12-20

**Authors:** Jia Cao, Ling Jin, Zi-Qi Yan, Xiao-Kai Wang, You-You Li, Zun Wang, Yi-Wei Liu, Hong-Ming Li, Zhe Guan, Ze-Hui He, Jiang-Shan Gong, Jiang-Hua Liu, Hao Yin, Yi-Juan Tan, Chun-Gu Hong, Shi-Kai Feng, Yan Zhang, Yi-Yi Wang, Lu-Yue Qi, Chun-Yuan Chen, Zheng-Zhao Liu, Zhen-Xing Wang, Hui Xie

**Affiliations:** 1 Department of Orthopedics, Movement System Injury and Repair Research Center, Xiangya Hospital, Central South University, Changsha, Hunan 410008 China; 2 Hunan Key Laboratory of Angmedicine, Changsha, Hunan 410008 China; 3 National Clinical Research Center for Geriatric Disorders, Xiangya Hospital, Central South University, Changsha, Hunan 410008 China

**Keywords:** Transdifferentiation, Mesenchymal stem cells, Regeneration

## Abstract

Endothelial cells (ECs) and bone marrow stromal cells (BMSCs) play crucial roles in supporting hematopoiesis and hematopoietic regeneration. However, whether ECs are a source of BMSCs remains unclear. Here, we evaluate the contribution of endothelial-to-mesenchymal transition to BMSC generation in postnatal mice. Single-cell RNA sequencing identifies ECs expressing BMSC markers *Prrx1* and *Lepr*; however, this could not be validated using *Prrx1-Cre* and *Lepr-Cre* transgenic mice. Additionally, only a minority of BMSCs are marked by EC lineage tracing models using *Cdh5-rtTA-tetO-Cre* or *Tek-CreERT2*. Moreover, *Cdh5*^+^ BMSCs and *Tek*^+^ BMSCs show distinct spatial distributions and characteristic mesenchymal markers, suggestive of their origination from different progenitors rather than CDH5^+^ TEK^+^ ECs. Furthermore, myeloablation induced by 5-fluorouracil treatment does not increase *Cdh5*^+^ BMSCs. Our findings indicate that ECs hardly convert to BMSCs during homeostasis and myeloablation-induced hematopoietic regeneration, highlighting the importance of using appropriate genetic models and conducting careful data interpretation in studies concerning endothelial-to-mesenchymal transition.

## Abstract (web summary)

Endothelial cells (ECs) and bone marrow stromal cells (BMSCs) support hematopoiesis and hematopoietic regeneration. Whether ECs are a source of BMSCs remains unclear. Here, using lineage tracing models the authors show that ECs are not a source of BMSCs during homeostasis and regeneration.

## Introduction

Endothelial cells (ECs) form the inner surface of the vascular system as a monolayer. They perform unique tissue-specific functions and demonstrate plasticity under certain conditions^1–3^. Hemogenic ECs in the embryonic yolk sac and dorsal aorta transdifferentiate into hematopoietic progenitors that produce blood cells throughout life^4^. Additionally, ECs in various organs can also give rise to mesenchymal cells, including smooth muscle cells (SMCs), pericytes (PCs), fibroblasts, and multipotent stem-like cells^5–11^. This endothelial-to-mesenchymal transition (EndoMT) contributes to muscle formation/regeneration and heart development^5–7^. However, it may also play a role in the pathogenesis of fibrotic disorders and cancer^8–11^.

It is generally accepted that in the process of EndoMT, ECs gradually lose endothelial markers while acquiring mesenchymal markers^5–11^. Consequently, mesenchymal cells expressing endothelial markers such as CD31 (PECAM1), CDH5 (VE-cadherin), TIE2 (TEK), and endomucin (EMCN), as well as ECs expressing characteristic markers of specific mesenchymal cell types, are commonly recognized as intermediates of EndoMT^5–11^. Additionally, Cre-expressing mouse models driven by *Cdh5* or *Tek* promoters and enhancers are often employed to genetically identify EC-derived cells, with inducible Cre models preferred to differentiate the prenatal and postnatal contributions of ECs^5–7,9^.

Conflicting reports exist on whether ECs serve as a source of bone marrow stromal cells (BMSCs), which contain skeletal stem cells (SSCs) and are vital for bone metabolism and hematopoietic cell maintenance^12–14^. Human embryonic stem cell differentiation assays have revealed the presence of endothelial markers on colonies that later develop into BMSC-like cells, indicating that BMSCs might originate from ECs^15,16^. A constitutively active *Cdh5-Cre* mouse strain has been reported to label approximately 40% of cultured BMSCs^17^. Additionally, subsets of bone marrow ECs have been identified to express BMSC markers *Prrx1* and *Lepr* in single-cell RNA sequencing (scRNA-seq) analysis^17^. In contrast, other studies have indicated that a different *Cdh5-Cre* strain and an inducible *Cdh5-CreERT2* strain fail to label BMSCs in scRNA-seq and immunostaining analyses^2,18,19^, while *Prrx1-Cre* and *Lepr-Cre* transgenic mice do not label bone marrow ECs in flow cytometry and immunostaining analyses^13,20^. Notably, EC-derived BMSCs have been reported to possess the potential to generate and regenerate the hematopoietic niche^17^. However, despite the observed increase in the number of ECs expressing certain mesenchymal markers following chemotherapy^17^, whether there is an actual increase in *Cdh5*-*Cre*-labeled BMSCs in this context remains unclear. It is essential to confirm the increase in the *Cdh5*-*Cre*-labeled BMSC population to validate the conversion of ECs to BMSCs.

In this study, we employed genetic BMSC and EC lineage tracing models, along with scRNA-seq, flow cytometry, and immunostaining techniques, to investigate whether ECs give rise to BMSCs in postnatal mice during homeostasis and hematopoietic generation induced by 5-fluorouracil (5-FU) treatment.

## Results

### Potential EndoMT intermediates detected by scRNA-seq

**Fig. 1:**
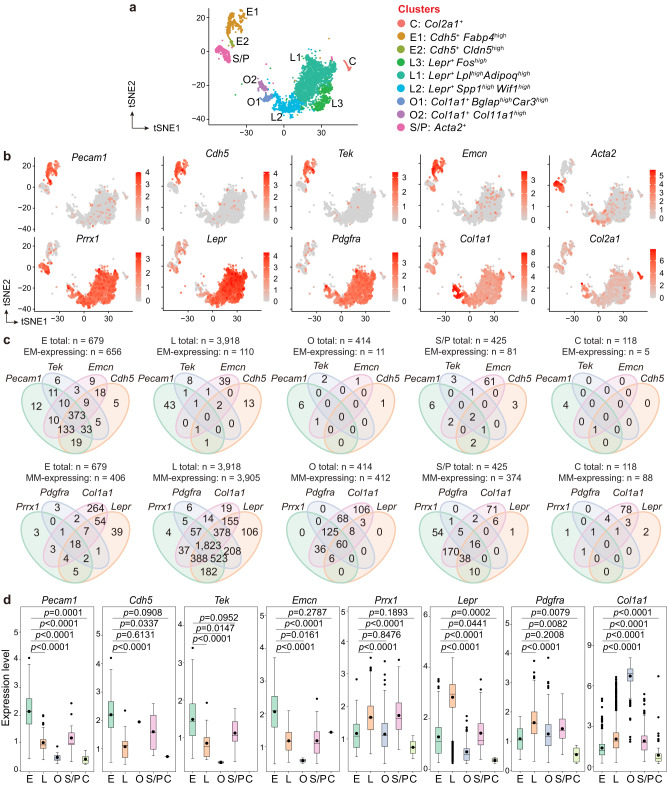
Potential intermediates of EndoMT are detected in postnatal bone marrow using scRNA-seq. **a** t-SNE visualization of EC and stromal cell clusters (*n* = 5554 ECs and stromal cells) in the scRNA-seq of collagenase-digested bone and bone marrow cells from 5-week-old wild-type mice (*n* = 3 mice). E ECs. L LEPR^+^ BMSCs. O osteolineage cells. C chondrolineage cells. S/P SMCs/PCs. **b**, **c** t-SNE diagrams showing the presence of endothelial and mesenchymal markers (**b**), and the Venn diagrams showing the co-expression of endothelial and mesenchymal markers (**c**) in ECs and stromal cell subtypes. **d** Boxplots showing the transcript levels of endothelial and mesenchymal markers in ECs and stromal cell subtypes expressing these markers (number of cells expressing endothelial/mesenchymal markers are shown in **c**). EM, endothelial marker. MM, mesenchymal markers. Boxplots display the following parameters: the median (middle line), the first and third quartiles (lower and upper edges of the “boxes”), the largest/smallest values no further than 1.5 times the distance between the first and third quartiles (upper/lower whiskers), data beyond the end of the whiskers (individually plotted dots), and the mean (small dots within “boxes”). Statistical significance was determined by two-tailed Wilcox rank-sum test. Source data are provided as a Source Data file.

First, to identify cells potentially undergoing EndoMT in postnatal bone marrow, we performed scRNA-seq on collagenase-digested bone and bone marrow cells from 5-week-old wild-type C57BL/6J mice. After excluding cells with known hematopoietic properties, a total of 5554 cells were obtained. These cells included ECs (*Cdh5*^+^*Pecam1*^+^, clusters E1 and E2) as well as the following stromal cell subtypes: LEPR^+^ BMSCs (*Lepr*^*+*^, clusters L1−L3), osteolineage cells (*Col1a1*^+^, clusters O1 and O2), chondrolineage cells (*Col2a1*^+^, cluster C), and SMCs/PCs (*Acta2*^+^, cluster S/P) (Fig. 1a and Supplementary Data 1).

As illustrated in the t-distributed stochastic neighbor embedding (t-SNE) diagrams, both E1 and E2 clusters were enriched in endothelial markers, including *Pecam1*, *Cdh5, Tek*, and *Emcn*. Within the stromal cell clusters, the majority (over 70−80%) of LEPR^+^ BMSCs and the O1 cluster of osteolineage cells expressed the mesenchymal markers *Prrx1* (encoding PRRX1, a mesenchymal transcript factor) and *Pdgfra* (encoding PDGFRα, a receptor for platelet-derived growth factor) (Fig. 1b and Supplementary Data 1). *Prrx1* was only found in 2.5% of chondrolineage cells and 25% of the O2 cluster of osteolineage cells (Fig. 1b and Supplementary Data 1), suggesting that these cells represented differentiated progenies of BMSCs that initially expressed but subsequently lost *Prrx1*^21^. Additionally, 69% of SMCs/PCs also expressed *Prrx1* (Fig. 1b). The mesenchymal marker *Lepr* (encoding the leptin receptor) was primarily observed in LEPR^+^ BMSCs, whereas *Col1a1* was enriched in osteolineage cells and also widely expressed in LEPR^+^ BMSCs (Fig. 1b and Supplementary Data 1).

Next, we analyzed the expression of endothelial markers in BMSCs and mesenchymal markers in ECs. The t-SNE diagrams illustrated the presence of *Pecam1*, *Cdh5, Tek*, and *Emcn* in small populations of cells within most stromal cell clusters (Fig. 1b and Supplementary Data 1). However, the Venn diagrams revealed that while these endothelial markers highly overlapped on ECs, they seldom overlapped on stromal cells (Fig. 1c). Individual endothelial markers were primarily detected in less than 2% of cells in the stromal cell subtypes, with the exception of *Emcn*, which was present in 15% of SMCs/PCs (Fig. 1c and Supplementary Data 1). As shown in boxplots, the average expression levels of endothelial markers were significantly lower in most stromal cell subtypes than in ECs (Fig. 1d).

In contrast to the small subsets of BMSCs that expressed endothelial markers, a relatively higher proportion of ECs were found to express mesenchymal markers. Specifically, *Prrx1* and *Lepr* were found in 5% and 19% of ECs, respectively; additionally, 5% of ECs presented *Pdgfra*, while a remarkable 52% of ECs expressed *Col1a1* (Fig. 1b and Supplementary Data 1). The Venn diagrams revealed that 62% of *Pdgfra*-expressing ECs also expressed *Prrx1*, while 80% of *Prrx1*-expressing ECs were also positive for *Lepr* (Fig. 1c). Boxplots demonstrated that the average transcript levels of *Prrx1*, *Lepr*, *Col1a1*, and *Pdgfra* were significantly lower in ECs than in the stromal cell subtypes expressing the respective mesenchymal markers (Fig. 1d).

The above results demonstrate the existence of an EC subset expressing various mesenchymal markers, and suggest that although *Pecam1*, *Cdh5, Tek*, and *Emcn* are expressed by subsets of BMSCs at low levels, individual BMSCs rarely co-express more than one of these endothelial markers. In line with these findings, pseudotime analysis between ECs and the stromal cell subtypes has revealed that along the differentiation trajectories, a subset of cells displayed a gradual decrease in endothelial markers and an increase in mesenchymal markers from endothelial branches to stromal cell branches (Supplementary Fig. 1a−d).

We also reanalyzed publicly available scRNA-seq datasets that characterized BMSCs from mice aged 5–22 weeks^19^, 8–12 weeks^22^, or 8–10 weeks^23^. In addition to the stromal cell subtypes identified in our scRNA-seq analysis, two reanalyzed datasets contained fibroblast clusters (*Ly6a*^*+*^*Pdgfra*^*+*^) (Supplementary Fig. 2a−c). The t-SNE diagrams of these datasets confirmed the presence of endothelial markers in stromal cells and mesenchymal markers in ECs, with frequencies similar to those observed in our scRNA-seq (Supplementary Fig. 2d−f and Supplementary Data 2). Additionally, stromal cells rarely co-expressed different endothelial markers and expressed endothelial markers at lower levels than ECs (Supplementary Fig. 3a, b). Individual endothelial markers were typically detected in fewer than 5% of the stromal cells, with the exception that *Emcn* was expressed in 40% of SMCs/PCs^19^ and *Tek* was present in 19% of endosteal fibroblasts^22^ (Supplementary Data 2). These findings indicate that potential intermediates of EndoMT are found in bone marrow at various postnatal stages in scRNA-seq analysis and that BMSCs often express single endothelial markers at low levels.

### EC subset with EndoMT-related genes identified via scRNA-seq

**Fig. 2:**
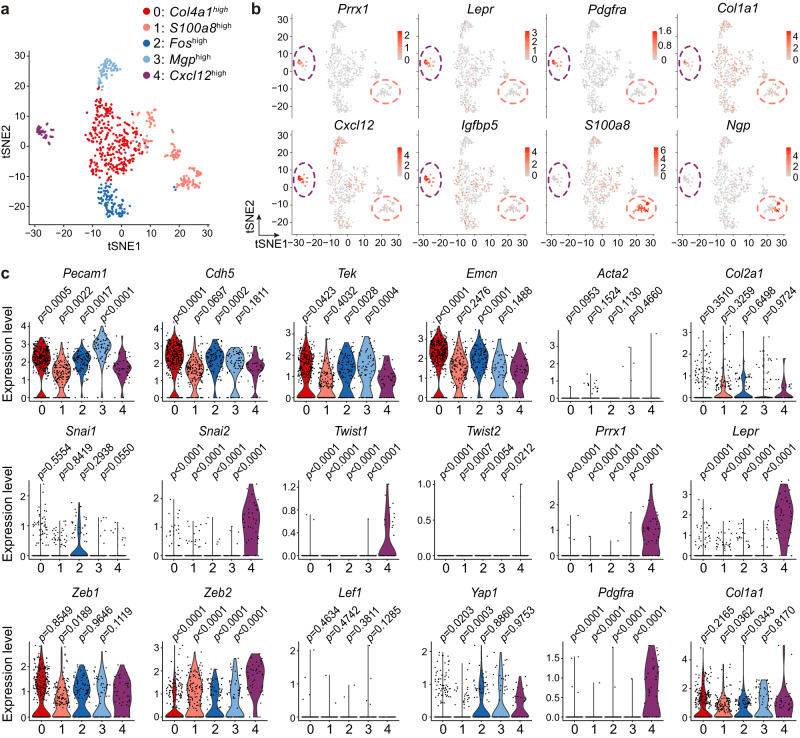
EC subset with EndoMT-related gene expression profiles is identified via scRNA-seq analysis. **a** t-SNE visualization of EC subclusters in the scRNA-seq of collagenase-digested bone and bone marrow cells. **b**, **c** t-SNE (**b**) and violin (**c**) plots showing the expression of endothelial markers, mesenchymal markers, and EndoMT-related transcript factors among the EC subclusters. Statistical significance was determined by two-tailed Wilcox rank-sum test.

To more specifically characterize ECs with mesenchymal features based on scRNA-seq data, we performed a re-clustering of ECs using higher resolution parameters. The results revealed that within the five EC subclusters (clusters 0−4) identified (Fig. 2a), EC subcluster 4 exhibited enrichment of mesenchymal markers, including *Prrx1*, *Lepr*, and *Pdgfra*, while *Col1a1* was similarly expressed across all EC subclusters (Fig. 2b). Notably, the top 10 marker genes of EC subcluster 4 highly overlapped with those of the L1 cluster of LEPR^+^ BMSCs (Supplementary Data 3). LEPR^+^ BMSCs are known to be the major source of hematopoietic niche factors in bone marrow^19,22^, and EC subcluster 4 also expressed high levels of these factors, such as *Cxcl12* and *Igfbp5* (Fig. 2b and Supplementary Data 3). On the other hand, EC subcluster 1 expressed several neutrophil marker genes^24^, including *S100a8* (encodes Calgranulin A) and *Ngp* (encodes neutrophilic granule protein) (Fig. 2b and Supplementary Data 3).

Furthermore, we analyzed the expression levels of endothelial markers, mesenchymal markers, and a set of genes typically upregulated in the process of EndoMT across the EC subclusters. Our analysis revealed that EC subcluster 4 exhibited significantly lower expression of *Pecam1*, *Cdh5, Tek*, and *Emcn* compared with EC subclusters 0 and 2, as well as significantly lower expression of *Pecam1* and *Tek* compared with EC subcluster 3 (Fig. 2c). However, the expression levels of endothelial markers were similar between EC subcluster 1 and 4 (Fig. 2c). The genes *Snai2*, *Twist1*, *Twist2*, and *Zeb2*, known to be key transcript factors that drive the process of EndoMT^25^, exhibited significantly higher expression in EC subcluster 4 compared with all other EC subclusters (Fig. 2c). These findings implied that, similar to embryonic bone marrow^17^, a subset of ECs with reduced levels of endothelial markers, increased levels of mesenchymal markers, and upregulated expression of EndoMT-related transcript factors can be observed in the scRNA-seq of postnatal bone marrow.

### EC-BMSC doublets as main source of BMSC marker-expressing ECs

Despite the above identification of ECs expressing *Prrx1* and *Lepr*, previous studies have reported that a *Lepr-Cre* mouse model does not label ECs in bone sections and a *Prrx1-Cre* mouse model marks minimal CD31^+^ cells in flow cytometry^13,20^. To validate the presence of *Prrx1*^+^ ECs and *Lepr*^+^ ECs, we mated *Prrx1-Cre* and *Lepr-Cre* strains with the *Rosa26-LSL-tdTomato* Cre reporter strain to examine Tomato^+^ ECs in double transgenic *Prrx1-Cre*;*Rosa26*^*LSL-tdTomato/+*^ (Prrx1-Cre;R26T) and *Lepr*^*Cre/+*^;*Rosa26*^*LSL-tdTomato/+*^ (Lepr-Cre;R26T) mice. In bone marrow, blood vessels mainly consist of arteries, arterioles, and sinusoids. Arteries and arterioles are positive for CD31 but negative for EMCN, while sinusoids express both markers. However, we did not observe any Tomato^+^ cells within CD31/EMCN^+^ ECs in tibia/femur sections and in vitro bone marrow EC cultures of 5-week-old Prrx1-Cre;R26T and Lepr-Cre;R26T mice (Supplementary Fig. 4a−d).

**Fig. 3:**
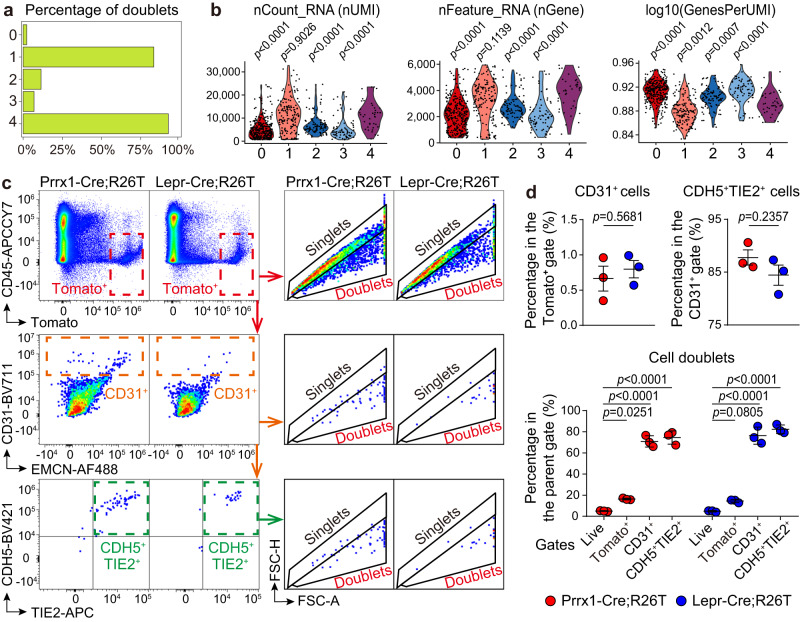
EC-BMSC heterotypic doublets are the predominant source of ECs expressing mesenchymal markers in scRNA-seq analysis. **a**, **b** Bar graph (**a**) and violin plots (**b**) showing the doublet ratios, nUMI, nGene, and log10(GenesPerUMI) among the EC subclusters. **c**, **d** Flow cytometry analysis illustrating the gating strategy (**c**) and quantification (**d**) of Tomato^+^ cells, Tomato^+^CD31^+^ cells, Tomato^+^CD31^+^CDH5^+^TIE2^+^ cells, and the doublet ratios of these cell populations in the live, Lin^−^CD45^−^ bone marrow cells of Prrx1-Cre;R26T (*n* = 3 biologically independent animals) and Lepr-Cre;R26T (*n* = 3 biologically independent animals) mice. Data represent the mean ± S.E.M. Statistical significance was determined by two-tailed Wilcox rank-sum test (**b**), two-tailed unpaired Student’s *t*-test or one-way ANOVA with Dunnett’s test (**d**). Source data are provided as a Source Data file.

To address the discrepancy between the identification of ECs expressing *Prrx1*/*Lepr* in scRNA-seq and the absence of Tomato^+^ ECs in the double transgenic models, we further analyzed other characteristics of the EC subclusters. Concerns have been raised regarding the presence of cell doublets in scRNA-seq analysis, which account for approximately 10% of cells in regular datasets and can increase further when numerous cells are obtained^26,27^. Heterotypic doublets formed by different cell types can create artificial intermediate cell states between these cell types and complicate pseudotime trajectory analysis^27,28^. We had initially excluded cell doublets from our scRNA-seq dataset by removing cells at the top quantiles of the number of genes (nGene) and the number of unique molecular identifiers (nUMI, i.e., total transcripts) detected per cell^17,19^. As this method may not effectively identify all cell doublets, we employed the scDblFinder package, which detects heterotypic doublets by creating artificial doublets and evaluating their prevalence in the neighborhood of each cell^26,28^. We found that the doublet ratios were 2−11% in EC subclusters 0, 2, and 3, but as high as 83% and 93% in EC subclusters 1 and 4, respectively (Fig. 3a). The nGene and nUMI parameters in EC subclusters 4 were significantly higher than those in EC subclusters 0, 2, and 3, and exhibited similar levels to those in EC subclusters 1 (Fig. 3b).

To investigate whether ECs expressing *Prrx1* and *Lepr* in scRNA-seq reflected EC-BMSC doublets, we performed flow cytometry analysis in which bone marrow samples of Prrx1-Cre;R26T and Lepr-Cre;R26T mice were prepared as the same as the scRNA-seq samples. Our results showed that in Prrx1-Cre;R26T and Lepr-Cre;R26T mice, only approximately 0.7% and 0.8%, respectively, of the total live, nonhematopoietic (Lineage(Lin)^−^CD45^−^) Tomato^+^ cells were CD31^+^ (Fig. 3c, d). Additionally, 85.8% and 84.4% of the CD31^+^ cells were CDH5^+^TIE2^+^ in Prrx1-Cre;R26T and Lepr-Cre;R26T mice, respectively (Fig. 3c, d), suggesting that these Tomato^+^CD31^+^ cells were bona fide ECs. However, when the CD31^+^ and CD31^+^CDH5^+^TIE2^+^ cell populations were analyzed for forward scatter area versus height (FSC-A/FSC-H) features^29^, nearly 80% of these cells were found to fall within the “doublet” gates rather than the “singlet” gates (Fig. 3c, d).

There may still be other possible reasons for the detection of ECs expressing *Prrx1* and *Lepr* in the scRNA-seq analysis. Notably, the *Lepr-Cre* utilized the *ObRb* splice that encodes the longest isoform of LEPR, while other splice variants may also be detected in scRNA-seq^13^. To examine the presence of *Prrx1* and *ObRb* in ECs, we performed RNA in situ hybridization (RNA-ISH) analysis with RNAscope probes against *Prrx1* (hybridizes with NM_011127.2, nucleotides 254–1726) and *Lepr* (hybridizes with NM_146146.2, nucleotides 3220–4109, which is on *ObRb* but no other *Lepr* splices) in tibia and femur sections of wild-type mice. We found that in addition to perivascular cells, *Prrx1* and *ObRb* were observed in a small number of CD31/EMCN^+^ ECs (Supplementary Fig. 4e). One possible explanation for this phenomenon could be that some ECs received these transcripts from exosomes of BMSCs, as donor cells could deliver mRNA encoding their characteristic markers to target cells via exosomes^30^. However, the expression of Cre recombinase and Tomato is induced only when *Prrx1* or *ObRb* is expressed within a specific cell itself.

### Minority of BMSCs labeled with EC tracing models

For the assessment of EndoMT, conducting EC lineage tracing experiments is crucial to evaluate the contribution of ECs, regardless of whether *Prrx1*^+^ ECs or *Lepr*^+^ ECs were detected or not. Therefore, we employed *Cdh5-rtTA-tetO-Cre* and *Tek-CreERT2* models and generated *Cdh5*^*rtTA-tetO-Cre/+*^;*Rosa26*^*LSL-tdTomato/+*^ (Cdh5-tetO-Cre;R26T) and *Tek-CreERT2*;*Rosa26*^*LSL-tdTomato/+*^ (Tek-CreERT2;R26T) mice. In these mice, *Cdh5*^+^ cells and *Tek*^+^ cells would exhibit Tomato fluorescence after doxycycline and tamoxifen treatment, respectively. Immunostaining analysis demonstrated that when the double transgenics were treated with doxycycline/tamoxifen from postnatal day 21 (P21) to P25 and examined on P30 (Supplementary Fig. 5a), *Cdh5*^+^ cells and *Tek*^+^ cells represented over 90% of CD31/EMCN^+^ ECs in tibia/femur sections and bone marrow EC cultures (Supplementary Fig. 5b−e). These findings indicated that both Cre models effectively labeled bone marrow ECs.

**Fig. 4:**
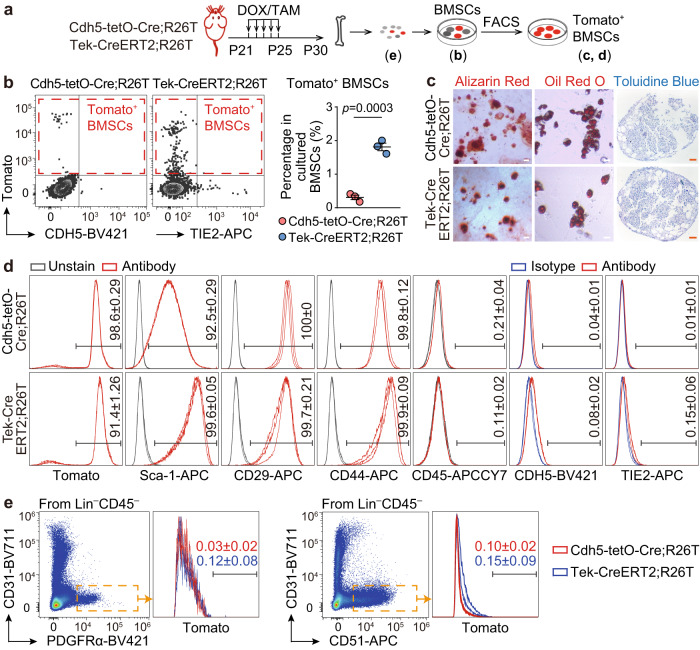
A small portion of BMSCs are labeled with EC lineage tracing models. **a** Diagrams of protocols. Notes on the timeline indicate the age of mice. **b** Flow cytometry analysis showing the percentage of Tomato^+^ cells within cultured BMSCs from Cdh5-tetO-Cre;R26T (*n* = 3 biologically independent animals) and Tek-CreERT2;R26T (*n* = 3 biologically independent animals) mice. **c**, **d** Analysis of osteogenic (Alizarin Red staining), adipogenic (Oil Red O staining), and chondrogenic (Toluidine Blue staining) differentiation capacities (**c**), repeated independently three times with similar results, as well as expression of mesenchymal, hematopoietic, and endothelial markers (**d**), numbers in plots indicate the percentage of cells that stained positive for antibodies in FACS-purified Tomato^+^ BMSCs from Cdh5-tetO-Cre;R26T (*n* = 3 biologically independent samples) and Tek-CreERT2-R26T (*n* = 3 biologically independent samples). **e** Percentage of Tomato^+^ cells in uncultured BMSCs from Cdh5-tetO-Cre;R26T (*n* = 3 biologically independent animals) and Tek-CreERT2-R26T (*n* = 3 biologically independent animals) mice in flow cytometry analysis. Scale bars: 50 µm. Data represent the mean ± S.E.M. Statistical significance was determined by two-tailed unpaired Student’s *t*-test. Source data are provided as a Source Data file.

Next, we examined *Cdh5*^+^ BMSCs and *Tek*^+^ BMSCs. When BMSCs were isolated by in vitro bone marrow culture, Tomato^+^ cells were present in the cultured BMSCs of both double transgenic models (Fig. 4a, b). Upon fluorescence-activated cell sorting (FACS), the purified Tomato^+^ cells exhibited the capability to differentiate into osteogenic, adipogenic, and chondrogenic lineages (Fig. 4c). Furthermore, the FACS-purified Tomato^+^ cells uniformly expressed mesenchymal markers Sca-1, CD29, and CD44 while being negative for the hematopoietic marker CD45 (Fig. 4d). Additionally, only a minor proportion of Tomato^+^ BMSCs were positive for CDH5 or TIE2 (Fig. 4b, d), suggesting transient expression of these endothelial markers in BMSCs. However, *Cdh5*^+^ BMSCs and *Tek*^+^ BMSCs constituted only approximately 0.3% and 1.9% of the total cultured BMSCs, respectively (Fig. 4b).

Furthermore, we analyzed *Cdh5*^+^ BMSCs and *Tek*^+^ BMSCs in uncultured bone marrow cells of Cdh5-tetO-Cre;R26T and Tek-CreERT2;R26T mice. Flow cytometry analysis revealed that both *Cdh5*^+^ and *Tek*^+^ cells were identified in the Lin^−^CD45^−^CD31^−^ population that expressed mesenchymal markers PDGFRα^31^ or CD51^32^ (Fig. 4e). However, *Cdh5*^+^ cells and *Tek*^+^ cells accounted for less than 0.5% of the Lin^−^CD45^−^CD31^−^PDGFRα^+^/CD51^+^ phenotypically defined BMSCs (Fig. 4e).

These findings reveal that EC lineage tracing models only label a minor proportion of BMSCs during postnatal stages, indicating that ECs are unlikely to be a significant source of BMSCs. Notably, the frequency of *Tek*^+^ BMSCs was significantly higher than that of *Cdh5*^+^ BMSCs (Fig. 4b). Considering that both Cre models effectively labeled bone marrow ECs (Supplementary Fig. 5b–e), BMSCs derived from ECs should have been equally marked by these two models, regardless of whether the EndoMT process was complete or partial^33^. Therefore, it is possible that certain *Tek*^+^ BMSCs were not derived from ECs.

### Distinct stromal subpopulations marked by EC tracing models

To further elucidate whether BMSCs expressing endothelial markers indeed originate from ECs, we examined the spatial distribution of *Cdh5*^*+*^ BMSCs and *Tek*^*+*^ BMSCs in tibia and femur sections of Cdh5-tetO-Cre;R26T and Tek-CreERT2;R26T mice. Due to a lack of highly sensitive and specific antibodies for immunostaining BMSCs^34^, we analyzed Tomato^+^ cells that were not luminal CD31/EMCN^+^ cells and did not display the morphology of a small number of *Cdh5*^+^/*Tek*^+^ hematopoietic cells (Supplementary Fig. 6a, b).

**Fig. 5:**
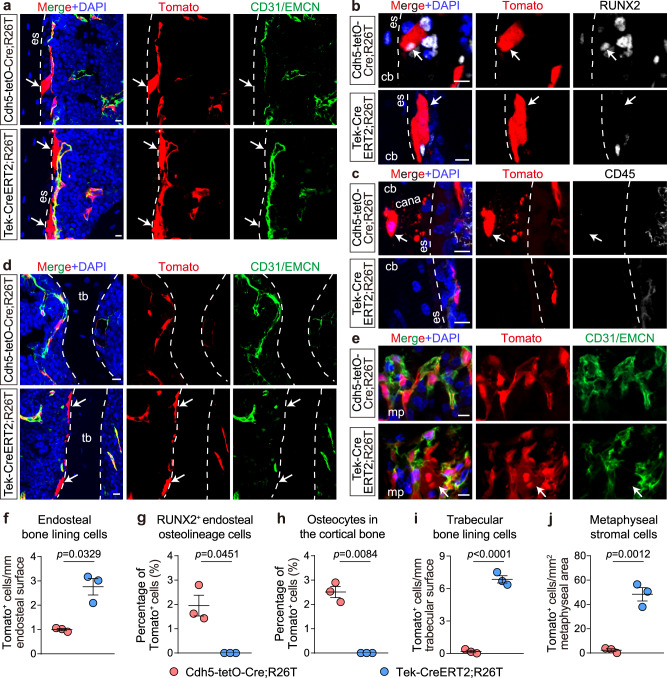
Different EC lineage tracing models label distinct stromal cell subpopulations in vivo. **a**–**e** Representative immunostaining images showing the differential labeling of endosteal bone-lining cells (**a**), RUNX2^+^ endosteal osteolineage cells (**b**), osteocytes in the cortical bone (**c**), trabecular bone-lining cells (**d**), and metaphyseal stromal cells (**e**) (arrows) by Cdh5-tetO-Cre;R26T (*n* = 3 biologically independent animals) and Tek-CreERT2-R26T (*n* = 3 biologically independent animals) models. **f**–**j** Quantification of Tomato^+^ endosteal bone-lining cells (**f**), RUNX2^+^ endosteal osteolineage cells (**g**), osteocytes in the cortical bone (**i**), trabecular bone-lining cells (**j**), and metaphyseal stromal cells (**i**) traced in the Cdh5-tetO-Cre;R26T and Tek-CreERT2-R26T models. Scale bars: 10 µm. Data represent the mean ± S.E.M. Statistical significance was determined by two-tailed unpaired Student’s *t*-test (**f**–**h** adjusted for unequal variances with Welch’s test). Source data are provided as a Source Data file.

In Cdh5-tetO-Cre;R26T mice, we identified a few *Cdh5*^+^ bone-lining cells at the endosteum, with an approximate frequency of 1 cell/mm endosteum in longitudinal sections (Fig. 5a, f). Immunostaining for the osteolineage marker RUNX2 revealed that approximately 2% of endosteal RUNX2^+^ cells were *Cdh5*^+^ (Fig. 5b, g). Additionally, *Cdh5*^+^ osteocytes with distinct canaliculi (characteristic structure of osteocytes) were observed, constituting approximately 2.5% of total osteocytes in the cortical bone (Fig. 5c, h). *Cdh5*^+^ stromal cells were barely detectable in other areas of the bone marrow cavity, including the metaphysis, trabecular bones, and diaphysis (Fig. 5d, e, i−j).

In Tek-CreERT2;R26T mice, we also observed *Tek*^+^ bone-lining cells at the endosteum (Fig. 5a). The frequency of these cells was approximately 2.8 cells/mm endosteum in longitudinal sections, which was significantly higher than that of *Cdh5*^+^ bone-lining cells (Fig. 5f). *Tek*^+^ endosteal bone-lining cells often exhibited a thin and elongated morphology and were nearly absent for the osteolineage marker RUNX2 (Fig. 5b, g), suggesting that they might correspond to the endosteal fibroblasts detected in the reanalyzed dataset^22^. However, *Tek*^+^ osteocytes were hardly detected in these mice (Fig. 5c, h). In other regions of the bone marrow cavity, a small population of trabecular bone-lining cells (Fig. 5d, i) and metaphyseal stromal cells (Fig. 5e, j) were also *Tek*^+^.

In a two-month chase after Cdh5-tetO-Cre;R26T and Tek-CreERT2;R26T mice were administered with doxycycline or tamoxifen, the distribution pattern of *Cdh5*^+^ BMSCs and *Tek*^+^ BMSCs remained unchanged (Supplementary Fig. 7a−e). These findings suggest that *Cdh5*^+^ BMSCs and *Tek*^+^ BMSCs represent distinct stromal cell subpopulations in bone marrow, indicating that they originate from different progenitors rather than CDH5^+^TIE2^+^ ECs.

### EMCN^+^ SMCs/PCs not labeled with EC tracing models

**Fig. 6:**
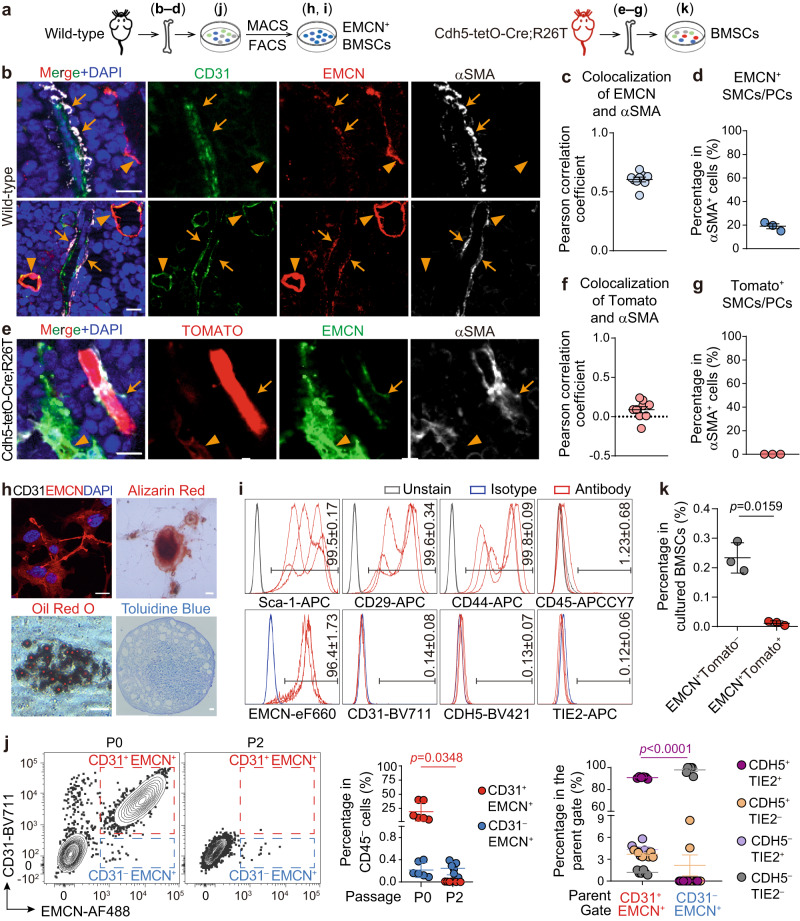
EMCN^+^ SMCs/PCs are not labeled by EC lineage tracing models. **a** Diagrams of protocols. **b**–**d**. Immunostaining analysis showing the presence of EMCN^low^ SMCs/PCs (arrows) surrounding or aligning with CD31^+^EMCN^−^ arteries and arterioles in tibia/femur sections of wild-type mice (*n* = 3 biologically independent animals). **e**–**g** Immunostaining analysis showing the absence of Tomato^+^ SMCs/PCs in tibia/femur sections of Cdh5-tetO-Cre;R26T mice (*n* = 3 biologically independent animals). **h**, **i** Analysis of osteogenic, adipogenic, and chondrogenic differentiation capacities (**h**, repeated independently three times with similar results), as well as expression of mesenchymal, hematopoietic, and endothelial markers (**i**) in MACS/FACS-purified EMCN^+^ BMSCs from wild-type mice (*n* = 3 biologically independent samples). **j** Flow cytometry analysis showing the CD31^+^EMCN^+^ and CD31^−^EMCN^+^ populations in passage 0 (P0, *n* = 6 biologically independent samples) and P2 (*n* = 6 biologically independent samples) bone marrow cells cultured in endothelial growth medium (EGM) and the percentage of CDH5^+^TIE2^+^ cells in the P0 EMCN^+^ populations. **k** Flow cytometry analysis of EMCN^+^ cells in EGM-cultured bone marrow cells from Cdh5-tetO-Cre;R26T mice (*n* = 3 biologically independent samples). Scale bars: 10 µm. Data represent the mean ± S.E.M. **c**, **f** Colocalization was quantified for EMCN^low^ SMCs/PCs and total SMCs/PCs, respectively (3 randomly chosen EMCN^low^ SMCs/PCs or total SMCs/PCs per biological replicate). Statistical significance was determined by two-tailed unpaired Student’s *t*-test adjusted for unequal variances with Welch’s test. Source data are provided as a Source Data file.

According to scRNA-seq analysis, subsets of SMCs/PCs expressed EMCN at low levels. Therefore, we investigated the presence of these cells in vivo and explored their lineage relationship with ECs (Fig. 6a). Immunostaining analysis on tibia/femur sections of wild-type mice revealed that, apart from sinusoids that expressed high levels of EMCN, subsets of αSMA^+^CD31^−^ cells surrounding or aligning with CD31^+^EMCN^−^ arteries/arterioles exhibited low levels of EMCN (Fig. 6b−d). However, in Cdh5-tetO-Cre;R26T mice, αSMA^+^ SMCs/PCs were uniformly negative for Tomato fluorescence (Fig. 6e−g), suggesting that both EMCN^+^ and EMCN^−^ SMCs/PCs were unlikely to have an endothelial origin.

In addition to identifying EMCN^+^ SMCs/PCs in vivo, we also identified EMCN^+^ BMSCs in bone marrow cultures. These cells were obtained by culturing and passaging bone marrow cells in an endothelial growth condition, followed by purifying EMCN^+^ cells combining magnetic-activated cell sorting (MACS) and FACS isolation (Fig. 6a). The purified EMCN^+^ cells showed osteogenic, adipogenic, and chondrogenic differentiation capacities (Fig. 6h) and uniformly expressed mesenchymal markers Sca-1, CD29, and CD44 (Fig. 6i). They were negative for hematopoietic and endothelial markers, including CD45, CD31, CDH5, and TIE2 (Fig. 6i).

To explain the absence of CD31^+^EMCN^+^ ECs following the MACS/FACS isolation of EMCN^+^ cells, we analyzed the bone marrow cells at different passages of cell culture. Our findings demonstrated that at passage 0, CD31^+^EMCN^+^ cells expressing CDH5 and TIE2 were readily detectable (Fig. 6j). Additionally, CD31^−^EMCN^+^ cells that lacked CDH5 and TIE2 were also observed (Fig. 6j). However, at passage 2, the frequency of CD31^+^EMCN^+^ cells significantly decreased, while CD31^−^EMCN^+^ cells were still detectable (Fig. 6j). This reduction in ECs during passaging reflects the previously reported challenges in establishing a sustainable culture of murine ECs^35^.

To investigate whether the cultured EMCN^+^ BMSCs were derived from ECs, we cultured bone marrow cells of Cdh5-tetO-Cre;R26T mice in endothelial growth conditions. Our results showed that more than 90% of the EMCN^+^ cells were negative for Tomato fluorescence (Fig. 6k), indicating that the majority of EMCN^+^ BMSCs were not derived from CDH5-expressing cells, including ECs.

### No increase in EC marker-expressing BMSCs post myeloablation

**Fig. 7:**
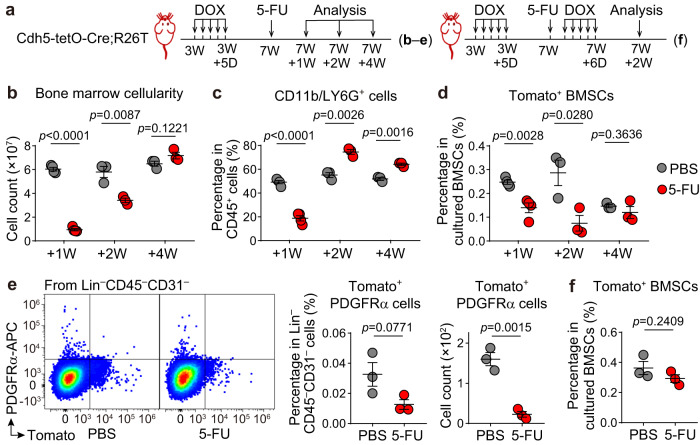
Endothelial marker-expressing BMSC population does not increase in myeloablation-induced hematopoietic regeneration. **a** Diagrams of protocols. Notes on the timeline indicate the age of mice. **b**–**d** Flow cytometry analysis depicting changes in bone marrow cellularity (**b**), the percentage of CD11b/LG6G^+^ myeloid cells in CD45^+^ hematopoietic cells (**c**), and the percentage of Tomato^+^ cells in cultured BMSCs (**d**) from Cdh5-tetO-Cre;R26T mice treated with PBS (*n* = 4, 3, 3 biologically independent animals at +1 W, +2 W, and +4 W, respectively) or 5-FU (*n* = 4, 3, 3 biologically independent animals at +1 W, +2 W, and +4 W, respectively). **e** Flow cytometry analysis showing the percentage and number of Tomato^+^PDGFRα^+^ cells within uncultured Lin^−^CD45^−^CD31^−^ cells from Cdh5-tetO-Cre;R26T mice treated with PBS (*n* = 3 biologically independent animals, at +2 W) or 5-FU (*n* = 3 biologically independent animals, at +2 W). **f** Analysis of Tomato^+^ BMSCs from Cdh5-tetO-Cre;R26T mice that administered an extra round of doxycycline after PBS (*n* = 3 biologically independent animals) or 5-FU (*n* = 3 biologically independent animals) treatment. Data represent the mean ± S.E.M. Statistical significance was determined by two-tailed unpaired Student’s *t*-test. Source data are provided as a Source Data file.

In postnatal bone marrow, both BMSCs and ECs are important for supporting hematopoiesis^19,22,23^. After chemotherapy, the depletion of myeloid cells triggers the regeneration of the hematopoietic system^36,37^. To investigate whether ECs contribute to hematopoietic regeneration by converting to BMSCs, we treated Cdh5-tetO-Cre;R26T mice with doxycycline at three weeks of age, followed by a single dose of 5-FU at 150 mg/kg body weight or equal volumes of PBS at seven weeks of age. Subsequently, we evaluated the frequencies of BMSCs derived from *Cdh5*^+^ cells at one, two, and four weeks after the administration of PBS or 5-FU (Fig. 7a).

One week post-administration of PBS/5-FU, a significant reduction in bone marrow cellularity and myeloid cell frequency was observed in the 5-FU group, indicating successful myeloablation (Fig. 7b, c). At this time point, the percentage of Tomato^+^ cells within total cultured BMSCs was found to be significantly lower in the 5-FU group than that in the PBS group (Fig. 7d). Two weeks after treatment, when hematopoietic cells began to recover (Fig. 7b, c)^36,37^, the percentage of Tomato^+^ BMSCs remained significantly lower in the 5-FU group (Fig. 7d). Four weeks after treatment, when the bone marrow cellularity was similar between the 5-FU-treated and control mice (Fig. 7b, c), there was no difference in the percentage of Tomato^+^ BMSCs between the two groups (Fig. 7d). At all analyzed time points, the percentage of Tomato^+^ BMSCs in both treatment groups remained consistently below 0.5% (Fig. 7d). Furthermore, flow cytometry analysis of uncultured bone marrow cells revealed that two weeks after treatment, Tomato^+^PDGFRα^+^ cells in the Lin^−^CD45^−^CD31^−^ population were also lower in the 5-FU-treated mice than in the control mice (Fig. 7e). These findings suggested that BMSCs derived from *Cdh5*^+^ cells did not increase during myeloablation-induced hematopoietic regeneration.

Following 5-FU treatment, ECs also undergo regression and regeneration^36^. It is possible that some of the regenerated ECs were derived from EC precursors that were not labeled during the original doxycycline treatment, which could potentially affect the evaluation of EC-to-BMSC conversions. To address this concern, we conducted an additional experiment in which Cdh5-tetO-Cre;R26T mice were administered with an extra round of doxycycline following PBS/5-FU treatment (Fig. 7a). In this experiment, the percentage of Tomato^+^ cells within total cultured BMSCs showed no differences between the 5-FU-treated and control mice two weeks after treatment (Fig. 7f). These findings provide further evidence that ECs do not give rise to BMSCs following 5-FU treatment.

## Discussion

In this study, we investigated the contribution of EndoMT to the generation of BMSCs in postnatal mice. Our results suggest that ECs are not a source of BMSCs during both homeostasis and myeloablation-induced hematopoietic regeneration, and that BMSCs expressing endothelial markers are distinct from BMSCs derived from ECs.

We report that ECs expressing mesenchymal markers *Prrx1* or *Lepr* were identified in scRNA-seq analysis, but could not be validated using *Prrx1-Cre* or *Lepr-Cre* transgenic mice. These cells predominantly represented EC-BMSC heterotypic doublets, which compromised the single-cell resolution of the scRNA-seq technique and led to the false identification of new cell types^26,27^. Notably, we observed significant upregulation of EndoMT-related transcript factors, namely *Snai2*, *Twist1*, *Twist2*, and *Zeb2*, in LEPR^+^ BMSCs compared to ECs (Supplementary Fig. 8a). This likely explains the higher expression of these genes in the *Prrx1*/*Lepr*-expressing EC subcluster compared to other EC subclusters (Fig. 2c). These findings underscore the importance of employing genetic models to validate new cell types present in scRNA-seq. The EC subcluster expressing neutrophil markers was also suggested to be primarily composed of heterotypic doublets as determined using the scDblFinder package (Fig. 3a). Consistent with this finding, there is no clear evidence that neutrophils are generated from ECs in postnatal bone marrow. In flow cytometry analysis, conventional gating approaches employing forward scatter features are not completely effective in eliminating heterotypic doublets^29^, and this may explain the observation of small fractions of *Prrx1*^+^ ECs and *Lepr*^+^ ECs within the “singlet” gates during analysis (Fig. 3c, d).

**Fig. 8:**
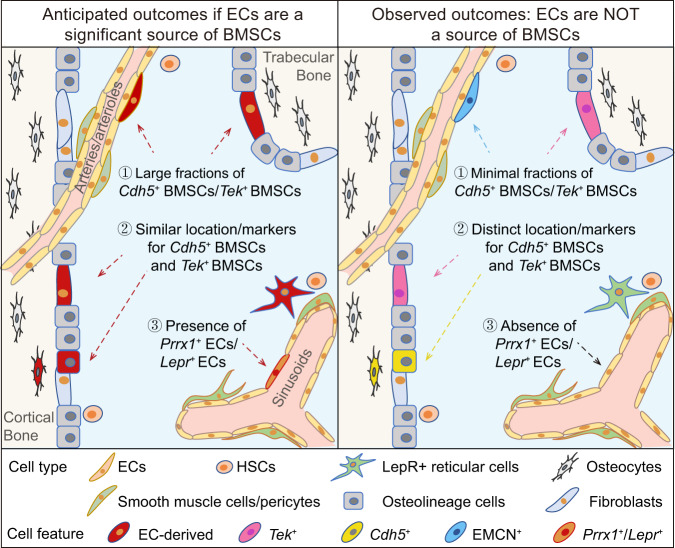
Graphical illustration of the anticipated and observed outcomes in bone marrow EndoMT investigations. The bone marrow cavity surrounding the cortical and trabecular bone is depicted. If ECs are a significant source of BMSCs, then large fractions of BMSCs should be marked by EC lineage tracing models *Cdh5-rtTA-tetO-Cre* and *Tek-CreERT2*, both of which effectively trace bone marrow ECs. Additionally, BMSCs derived from ECs should be similarly marked by the two EC lineage tracing models. Moreover, subsets of ECs may express mesenchymal markers such as PRRX1 and LEPR. However, the observed results showed that only a minor fraction of BMSCs were *Cdh5*^+^ or *Tek*^+^. Additionally, *Cdh5*^+^ BMSCs and *Tek*^+^ BMSCs displayed different frequencies, spatial distribution and characteristic mesenchymal markers. Furthermore, *Prrx1*^+^ ECs and *Lepr*^+^ ECs were not present.

BMSCs are composed of heterogeneous cell subpopulations that exhibit various characteristic markers and spatial distributions^38,39^. We demonstrate herein that EC lineage tracing models only labeled a minor fraction of BMSCs during homeostasis and myeloablation-induced hematopoietic regeneration. Furthermore, by demonstrating the effectiveness of the EC lineage tracing models in labeling bone marrow ECs and observing that *Cdh5*^+^ BMSCs and *Tek*^+^ BMSCs constituted two distinct stromal cell subpopulations, we provide evidence that *Cdh5*^+^ BMSCs and *Tek*^+^ BMSCs are not derived from ECs. The low frequencies of BMSCs expressing endothelial markers, together with their lack of endothelial lineage relationships, strongly suggest that ECs are not a source of BMSCs (illustrated in Fig. 8).

During embryonic development, the bone marrow vascular network forms at approximately embryonic (E) day 14.5^40^. We found that in Cdh5-tetO-Cre;R26T mice administered with doxycycline from E14.5 to E18.5, only approximately 1.5% of cultured BMSCs from the E18.5 mice were *Cdh5*^+^ (Supplementary Fig. 9a, b). Although we did not specifically examine the lineage origin of these *Cdh5*^*+*^ BMSCs, the low frequencies of these cells suggested that ECs were unlikely to be a significant source of BMSCs at the embryonic stage.

In addition to *Cdh5*^+^ BMSCs and *Tek*^+^ BMSCs, we also identified EMCN^+^ BMSCs that were not derived from ECs. The frequency of EMCN^+^ BMSCs was very low in cultured bone marrow cells (Fig. 6j). However, their presence should still be considered in studies involving ECs. For instance, when isolating H-type (CD31^high^EMCN^high^) ECs that play a crucial role in bone metabolism^2,41–43^, EMCN^+^ BMSCs may constitute the majority of EMCN^+^ cells purified through MACS or FACS (Fig. 6i). Therefore, it is essential to include an analysis of EMCN^+^ BMSCs during the isolation of EMCN^+^ ECs to prevent any erroneous characterization of EMCN^+^ BMSCs as ECs.

Various hematopoietic cells express low levels of CD31 (Supplementary Fig. 6a)^34^; therefore, we were unable to confidently identify CD31^+^ BMSCs in tibiae or femurs. Additionally, although our findings demonstrate that ECs are not a source of BMSCs during both homeostasis and myeloablation-induced hematopoietic regeneration, we cannot exclude the possibility that ECs may differentiate into BMSCs under other conditions. Considering the presence of BMSCs expressing endothelial markers but not derived from ECs, it is crucial to utilize appropriate lineage tracing models and conduct unbiased data interpretation in future EndoMT studies in bone marrow. Indeed, some researchers have warned that cell lineage analysis is complicated when a marker gene is simultaneously expressed by cell populations that do not convert into one another^44–46^. The development of EC-targeting models incorporating both the Cre–loxP and Dre–rox recombination systems^46^ could potentially provide more precise endothelial lineage analysis, as BMSCs seldom express more than one endothelial marker (Fig. 1c). The function of BMSCs expressing endothelial markers and the mechanism underlying their expression of endothelial markers require further investigation in future research.

## Methods

### Animal experiments and genetically modified mice

All experiments were performed according to the ethics approvals of the Institutional Animal Care and Use Committee of Central South University, Changsha, Hunan, China (Approval#: 2020sydw0800). Mice were maintained at a maximum of five mice per cage under a standard 12 h light/dark cycle, and had access to food and water *ad libitum*.

The following strains were used: C57BL/6J, B6.Cg-Tg(Prrx1-cre)1Cjt/J^47^ (The Jackson Laboratory), B6.129(Cg)-Leprtm2(cre)Rck/J^48^ (The Jackson Laboratory), B6.Cg-Gt(ROSA)26Sortm9(CAG-tdTomato)Hze/J^49^ (The Jackson Laboratory), B6.129S-Cdh5tm1(rtTA-tetO-Cre)Smoc (Shanghai Model Organisms Center, NM-KI-18006), B6.Cg-Tg(Tek-cre/ERT2)1Arnd/ArndCnrm^50^ (EMMA).

Pooled sample from both male and female mice were used in the FACS experiments and male mice were used in other experiments at the indicated postnatal ages. To generate Prrx1-Cre;R26T and Tek-CreERT2;R26T double transgenic mice, male *Prrx1-Cre* or *Tek-creERT2* mice were bred with female *Rosa26-LSL-tdTomato* mice. In the tamoxifen treatment, mice were intraperitoneally injected with tamoxifen (Sigma‒Aldrich, T5648) at a dose of 75 mg/kg body weight for 5 consecutive days. For doxycycline treatment, juvenile mice and pregnant dams were orally administered with doxycycline (MCE, HY-N0565B) in their drinking water^51^ (0.2 g doxycycline and 2 g sucrose per 100 mL water) for 5 and 4 consecutive days, respectively. In the 5-FU/PBS treatment, mice received 5-FU (Sigma‒Aldrich, F6627) at a dose of 150 mg/kg body weight or equal volumes of PBS for one dose via the intraperitoneal route.

### Isolation and culture of BMSCs

Protocols for isolating BMSCs from mouse bone marrow^52^ and compact bone^53^ were combined with some modifications. Briefly, tibiae and femurs were collected from euthanized mice and cleaned of attached muscles, connective tissues, and the epiphysis in PBS supplemented with 2% FBS. The cleaned bones were then cut into small pieces with scissors in stromal cell growth medium composed of αMEM supplemented with 15% qualified FBS (Gibco, 12664-025). Subsequently, the mixtures of bone chips and bone marrow were plated in tissue culture vessels. After 16–24 h, the culture medium was aspirated, and the cells were gently washed with PBS before being resupplied with fresh growth medium. The adherent cells were cultured for at least 2 passages to eliminate ECs and hematopoietic cells^52^ before being subjected to further analyses.

### Purification of Tomato^+^ BMSCs using FACS

To purify Tomato^+^ BMSCs from Cdh5-tetO-Cre;R26T or Tek-CreERT2;R26T mice, cultured BMSCs were detached from culture vessels using accutase (Gibco), passed through 40 μm cell strainers, and centrifuged at 300 × *g* for 5 min. The pellet was resuspended in PBS containing 2% FBS, and the Tomato^+^ cells were analyzed in the PE channel of the FACSAria II Cell Sorter (BD Biosciences). Single, viable Tomato^+^ cells were then sorted into PBS containing 10% FBS. The sorted cells were centrifuged at 300 × *g* for 5 min and plated onto tissue culture vessels in stromal cell growth medium.

### Osteogenic, adipogenic, and chondrogenic differentiation of cultured BMSCs

BMSCs in culture were subjected to osteogenic, adipogenic, or chondrogenic differentiation using the respective differentiation medium (Cyagen Biosciences) for up to 3 weeks following the manufacturer’s instructions. The differentiated cells were stained with Alizarin Red, Oil Red O, and Toluidine Blue, respectively.

### Isolation and culture of bone marrow ECs

Tibiae and femurs obtained from euthanized mice were cleaned of attached muscles, connective tissues, and the epiphysis. The cleaned bones were then cut into small pieces in digestion medium composed of αMEM supplemented with 2% FBS, 1 mg/mL type II collagenase (Gibco, 17101015), 1 mg/mL Dispase II (Sigma‒Aldrich, 04942078001), and 1 mg/mL DNase (Sigma‒Aldrich, 10104159001). The mixtures of bone chips and bone marrow were incubated in an orbital shaker at 120 r.p.m., 37 °C for 45 min. Subsequently, the samples were filtered through cell strainers and centrifuged, and the pellet was plated onto tissue culture vessels coated with collagen I (from rat tail, BD biosciences, 354236) in endothelial growth medium (EGM, Lonza, CC-3202). After 16–24 h, the culture medium was aspirated, and the cells were gently washed with PBS. The adherent cells containing ECs, hematopoietic cells, and BMSCs were subjected to flow cytometry or immunostaining analysis. Alternatively, the adherent cells were resupplied with fresh EGM to subsequently enrich for EMCN^+^ BMSCs.

### Purification of EMCN^+^ BMSCs using MACS and FACS

To isolate EMCN^+^ BMSCs, bone marrow cells cultured in EGM were detached from culture vessels using accutase. The cells were then passed through cell strainers, centrifuged, and resuspended in PBS containing 2% FBS. Subsequently, an EMCN-PE antibody (Santa Cruz, sc-65495) and anti-PE microbeads (Miltenyi Biotec, 130-048-801) were used to purify EMCN^+^ cells following the manufacturer’s instructions. The MACS-purified EMCN^+^ cells were immortalized with the SV40T antigen^54^ constructed in a recombinant lentivirus vector (Shanghai GeneChem Co., Ltd.). The immortalized cells were propagated for a few passages and then stained with the EMCN-PE antibody (eBioscience, 12-5851-82) to enrich for EMCN^+^ cells by FACS for 3 rounds.

### Flow cytometry

#### Sample preparation for uncultured bone marrow cells

Tibiae and femurs were collected and cleaned as described above and cut into small pieces in the digestion medium. The samples were then incubated in an orbital shaker at 120 r.p.m., 37 °C for 45 min, filtered through cell strainers, and centrifuged. The pellet was resuspended and subjected to flow cytometry analysis. Alternatively, the resuspended cells were depleted for lineage-positive cells using a lineage cell depletion kit (MiltenyiBiotec, 130-090-858) as per the manufacturer’s instructions before flow cytometry analysis.

#### Sample preparation for cultured bone marrow cells

The culture medium was aspirated, and the cells were washed with PBS. Subsequently, accutase was added to the culture vessels to detach the cells while preserving their endothelial markers. The detached cells were then collected and used for subsequent analyses.

#### Flow cytometry analysis and FACS

Cell suspensions were blocked with CD16/32 (clone 93, Biolegend, 101320, 1:200) at room temperature for 10 min and stained with the following antibodies at room temperature for 30 min: CD31-BV711 (clone 390, Biolegend, 303136, 1:100), EMCN-AF488/eFluor660 (clone V.7C7, e-Bioscience, 50-5851-82/53-5851-82, 1:100), TIE2-APC (clone TEK4, e-Bioscience, 17-5987-82, 1:100), CDH5-BV421 (clone 11D4.1, BD OptiBuild, 747749, 1:100), PDGFRα-APC/BV421 (clone APA5, BD Pharmingen, 562777/562774, 1:100), CD51-APC (clone RMV-7, Elabscience, E-AB-F1235E, 1:100), Sca-1 (clone D7, Biolegend, 108111, 1:100), CD44-APC (clone IM7, Biolegend, 103011, 1:100), CD29-APC (clone HMβ1-1, Biolegend, 102215, 1:100), CD45-PercpCy5.5/APC-CY7 (clone 30-F11, BD Pharmingen, 550994/557659, 1:100), Lineage cocktail-PercpCy5.5 (including Ly76, Ly-6G/Ly-6C, B220, CD11b, CD3e, clones TER119, RB6-8C5, RA3-6B2, M1/70, and 145-2C11, respectively, BD Pharmingen, 561317, 1:50), CD71-PercpCy5.5 (clone C2, BD Pharmingen, 562858, 1:100), CD19-APC (clone 1D3, eBioscience, 17-0193-80, 1:200), CD3-APC (clone 17A2, Cell Signaling Technology, 24265s, 1:200), Ly-6G-APC (clone 1A8, BD Pharmingen, 560599, 1:100), CD11b-FITC (clone M1/70, BD Pharmingen, 557396, 1:100), BV711 rat IgG2a κ isotype control (clone RTK2758, Biolegend, 400551, 1:100), BV421 rat IgG2a κ isotype control (clone R35-95, BD Horizon, 562602, 1:100), eFluor660 rat IgG2a κ isotype control (clone eBR2a, e-Bioscience, 50-4321-82, 1:100), APC rat IgG1 κ isotype control (clone eBRG1, e-Bioscience, 17-4301-82, 1:100). Cells were then washed with PBS and centrifuged. The pellet was resuspended in PBS supplemented with 2% FBS and subjected to flow cytometry analysis. To identify singlet cells, a gate was drawn based on the forward scatter area (FSC-A) versus forward scatter height (FSC-H) or forward scatter width (FSC-W) features, and dead cells were distinguished using Zombie Aqua dye (Biolegend, 423101, 1:1000).

#### Equipment

Flow cytometry analyses were performed on FACSCanto II Cell Analyzer (BD Biosciences) and Aurora Analyzer (Cytek Biosciences). Cell sorting was carried out using the FACSAria II Cell Sorter (BD Biosciences). Data were collected using Flowjo CE (Tree Star) or FACS DIVA software (6.1.3, BD Biosciences). The raw data were analyzed by FlowJo software (V10, Tree Star).

### Single-cell RNA sequencing and analysis

#### Sample preparation

Tibiae and femurs were collected from 5-week-old wild-type mice (*n* = 3 mice), cleaned of attached muscles, and cut into small pieces in the digestion medium. The released bone marrow (bm) and bone fragments (bo) were treated as separate samples and incubated in an orbital shaker at 120 r.p.m., 37 °C for 45 min. Subsequently, the samples were filtered through cell strainers and centrifuged. For the bm sample, lineage-positive cells were depleted using MACS (MiltenyiBiotec, 130-090-858), and then Lin^−^CD45^−^CD71^−^CD3^−^CD19^−^ cells were sorted using FACS. For the bo sample, erythrocytes were lysed (ThermoFisher Scientific, A1049201) and Lin^−^CD45^−^CD71^−^CD3^−^CD19^−^ cells were sorted using FACS.

#### Single-cell library preparation, sequencing, and analysis

Single-cell mRNA libraries were prepared for sequencing using the Chromium Next GEM Single Cell 3’ GEM, Library & Gel Bead Kit v3.1 (#1000121). The samples were sequenced using an Illumina NovaSeq 6000. Reads from scRNA-seq were aligned to mm10 and collapsed into UMI counts using 10x Genomics Cell Ranger software (version 4.0.0) with default parameters. Further analyses were performed using Seurat 3.0 in the R statistical language.

For quality control, the following cells were excluded from analysis: (1) Cells with more than 20% mitochondrial gene expression. (2) Cells in the top 2% quantile of nGene and nUMI. (3) Cells with the value of log10(GenesPerUMI) no more than 0.8. (4) Hematopoietic clusters and small clusters without clear characteristics of BMSCs or ECs. A total of 5554 cells were included for analysis. The re-clustering of ECs was performed using a resolution of 0.1. Doublets ratios in the EC subclusters were analyzed using the scDblFinder package (v.1.8.0) with default parameters^26^.

When cells were fractioned into two subsets, one positive for a target gene and one negative for the same gene, the cells with gene reads greater than zero were defined as positive, otherwise as negative.

Pseudotime analysis were performed between the EC clusters and each BMSC subtypes using the Monocle package^55^. We selected the top 1000 significantly differentially expressed genes as the ordering genes for the trajectory reconstruction.

All plots were generated using the ggplot2 and VennDiagram packages in R (4.0.2). Boxplots are displayed as follows: the median (middle line), the first and third quartiles (lower and upper edges of the “boxes”), the largest/smallest values no further than 1.5 times the distance between the first and third quartiles (upper/lower whiskers), data beyond the end of the whiskers (individually plotted dots), and the mean (small dots within “boxes”).

#### Reanalysis of scRNA-seq datasets from published literature

The original datasets were downloaded from the NCBI GEO database, and Cell Ranger software was used to conduct preliminary data analysis and generate the gene expression matrix. Following quality control, clusters were identified and named based on the original literature with some modifications. Hematopoietic clusters and small clusters without clear characteristics of BMSCs or ECs were excluded from analysis or visualization. The data were subsequently analyzed similarly to the scRNA-seq analysis of the bo/bm samples.

### Immunofluorescence assay and image acquisition

#### Immunostaining of tibia/femur sections

Tibiae and femurs were collected and fixed in 4% PFA at 4 °C for 24 h. After fixation, the samples were decalcified in 0.5 M EDTA (pH = 7.4) at 4 °C with constant shaking for 1–3 days and immersed in a 30% sucrose solution for 24 h before being embedded in O.C.T. Cryostat sections were generated at a thickness of 10–20 μm. These sections were rehydrated in PBS, blocked with PBS containing 5% donkey serum or 4% BSA at room temperature for 30 min, and then probed with primary antibodies diluted in the blocking solution overnight at 4 °C. After removing the primary antibodies, the sections were washed in PBS and stained with secondary antibodies for 45 min at room temperature. The nuclei were counterstained with DAPI (Vector) before mounting the sections with coverslips.

#### Immunostaining of cultured cells

The cells were plated on coverslips and allowed to grow before being fixed with 4% PFA at room temperature for 30 min. Subsequently, the coverslips were blocked with PBS containing 5% donkey serum at room temperature for 30 min. The cells were then incubated with primary antibodies overnight at 4 °C. Following the removal of the primary antibodies, the sections were washed in PBS and stained with secondary antibodies for 45 min at room temperature. The nuclei were counterstained with DAPI before mounting the coverslips on glass slides.

The following primary antibodies were used for immunostaining: CD31 (Abcam, ab28364, 1:100 for coverslips or R&D Systems, FAB3628G, 1:100 for bone sections), Endomucin (Santa Cruz, sc-65495, 1:200), alpha smooth muscle actin (Abcam, ab124964, 1:400), CD45 (BD Pharmingen, 557659, 1:200), and RUNX2 (Cell Signaling Technology, 12556 S, 1:400).

The following secondary antibodies were used in immunostaining (all obtained from Jackson ImmunoResearch): donkey anti-rabbit Alexa Fluor 488 (711-545-152, 1:400) and Alexa Fluor 647 (711-605-152, 1:400); donkey anti-rat Alexa Fluor 488 (712-545-150, 1:400), Alexa Fluor 594 (712-585-150, 1:400), and Alexa Fluor 647 (712-605-150, 1:400); and donkey anti-goat Alexa Fluor 488 (705-545-147, 1:400).

#### Quantification of colocalization in immunostaining analysis

In wild-type mice, we quantified the colocalization of EMCN and αSMA in EMCN^+^αSMA^+^ cells that surrounded or aligned with arteries or arterioles. Similarly, in Cdh5-tetO-Cre;R26T mice, we evaluated the colocalization of Tomato fluorescence and αSMA in αSMA^+^ cells surrounding or aligning with arteries/arterioles. Single channel images were generated using ZEN software (2.3, Zeiss) and analyzed with the Colocalization Finder tool in ImageJ software (1.54f, NIH, Bethesda, MD, USA). The Pearson correlation coefficient (PCC) was calculated between EMCN and αSMA, as well as Tomato fluorescence and αSMA. The PCC ranges from −1 to 1, with a value of 1 indicating a perfect linear relationship between the distributions of the two probes, −1 indicating a perfect inverse relationship, and 0 indicating an uncorrelated distribution of the probes^56^.

#### RNAscope ISH analysis

Tibiae and femurs were collected from euthanized mice and fixed with 4% PFA at 4 °C for 24 h. Decalcification was then performed using fast decalcification buffer (Beijing Pursuit Bio Co., Ltd.) at 4 °C with constant shaking for 20 h. After that, the samples were dehydrated in a series of ethanol solutions and cleared with xylene. Subsequently, the samples were embedded in paraffin wax to generate sections at a thickness of 10 μm. These sections were deparaffinized and rehydrated before undergoing ISH using the RNAscope® Multiplex Fluorescent Reagent Kit v2 following the manufacturer’s protocol. The target probes used in ISH were *Prrx1* (ACD 485231-C2, hybridizing with NM_011127.2, nucleotides 254–1726) and *Lepr* (ACD 471171, hybridizing with NM_146146.2, nucleotides 3220–4109). The mRNA signals were detected with opal570 and opal690, respectively. A probe against the bacterial *dapB* gene was used as a negative control. The sections were then subjected to immunostaining with CD31 and EMCN antibodies as described above.

#### Image acquisition

Images of immunostaining for cultured cells or bone sections were acquired using a Zeiss Axio Imager M2 microscope equipped with an ApoTome.2 system. Images of Alizarin Red, Oil Red O, and Toluidine Blue staining were acquired using an Olympus CX31 optical microscope. ZEN (2.3) and ImageJ (1.54f) software were used for image processing.

### Statistics & Reproducibility

The results are presented as the mean ± S.E.M. Differences between experimental groups were evaluated by two-tailed Student’s *t*-test (adjusted for unequal variances with Welch’s test where appropriate), one-way ANOVA, or two-tailed Wilcox rank-sum test, as indicated in the figure legends. GraphPad Prism software (v8.4) or R (4.0.2) was used for statistical analyses. *p* value less than 0.05 was considered statistically significant. No statistical method was used to predetermine the sample size. Required experimental sample sizes were estimated based on previous established protocols in the field. The experiments were not randomized, and the investigators were not blinded to the experimental allocation or outcome assessment.

### Reporting summary

Further information on research design is available in the Nature Portfolio Reporting Summary linked to this article.

### Supplementary information

Supplementary Information

Description of Additional Supplementary Files

Supplementary Data 1

Supplementary Data 2

Supplementary Data 3

Reporting Summary

### Source data

Source Data

## Data Availability

The raw scRNA-seq data are deposited in the NCBI GEO under accession code GSE168333. The reanalyzed datasets were obtained from the GEO database under the following accession codes: GSM2915578, GSM2915579^19^, GSE122465^22^, GSM3674224, GSM3674225, GSM3674226, GSM3674227, GSM3674228, and GSM3674229^23^. All other data are available within the article, Supplementary Information file, Source data file, or from the corresponding authors upon reasonable request. Source data are provided with this paper.

## Code availability

The scRNA-seq analysis in this study was conducted using publicly available codes: Seurat 3.0R package (https://satijalab.org/seurat/).
