# Supplementary material for: Reassessing endothelial-to-mesenchymal transition in mouse bone marrow: insights from lineage tracing models
Source: Nat Commun. 2023 Dec 20;14:8461. doi: 10.1038/s41467-023-44312-w (PMC10733381; doi:10.1038/s41467-023-44312-w)
Supplement: Supplementary file 1 — Supplementary Information [file 41467_2023_44312_MOESM1_ESM.pdf]

Supplementary Information

Reassessing endothelial-to-mesenchymal transition in mouse bone marrow: Insights from lineage tracing models

Jia Cao[1,2,3], Ling Jin[1,2], Zi-Qi Yan[1], Xiao-Kai Wang[1], You-You Li[1,2], Zun Wang[1], Yi-Wei Liu[1,2], Hong-Ming Li[1,2], Zhe Guan[1,2], Ze-Hui He[1,2], Jiang-Shan Gong[1,2], Jiang-Hua Liu[1], Hao Yin[1,2], Yi-Juan Tan[1,2], Chun-Gu Hong[1,2], Shi-Kai Feng[1], Yan Zhang[1], Yi-Yi Wang[1,2], Lu-Yue Qi[1], Chun-Yuan Chen[1,2,3], Zheng-Zhao Liu[1,2,3], Zhen-Xing Wang[1,2,3*], Hui Xie[1,2,3*]

[1]Department of Orthopedics, Movement System Injury and Repair Research Center, Xiangya Hospital, Central South University, Changsha, Hunan 410008, China.

[2]Hunan Key Laboratory of Angmedicine, Changsha, Hunan 410008, China.

[3]National Clinical Research Center for Geriatric Disorders, Xiangya Hospital, Central South University, Changsha, Hunan 410008, China.

*Correspondence to: Hui Xie (huixie@csu.edu.cn), Zhen-Xing Wang (wangzx@csu.edu.cn).

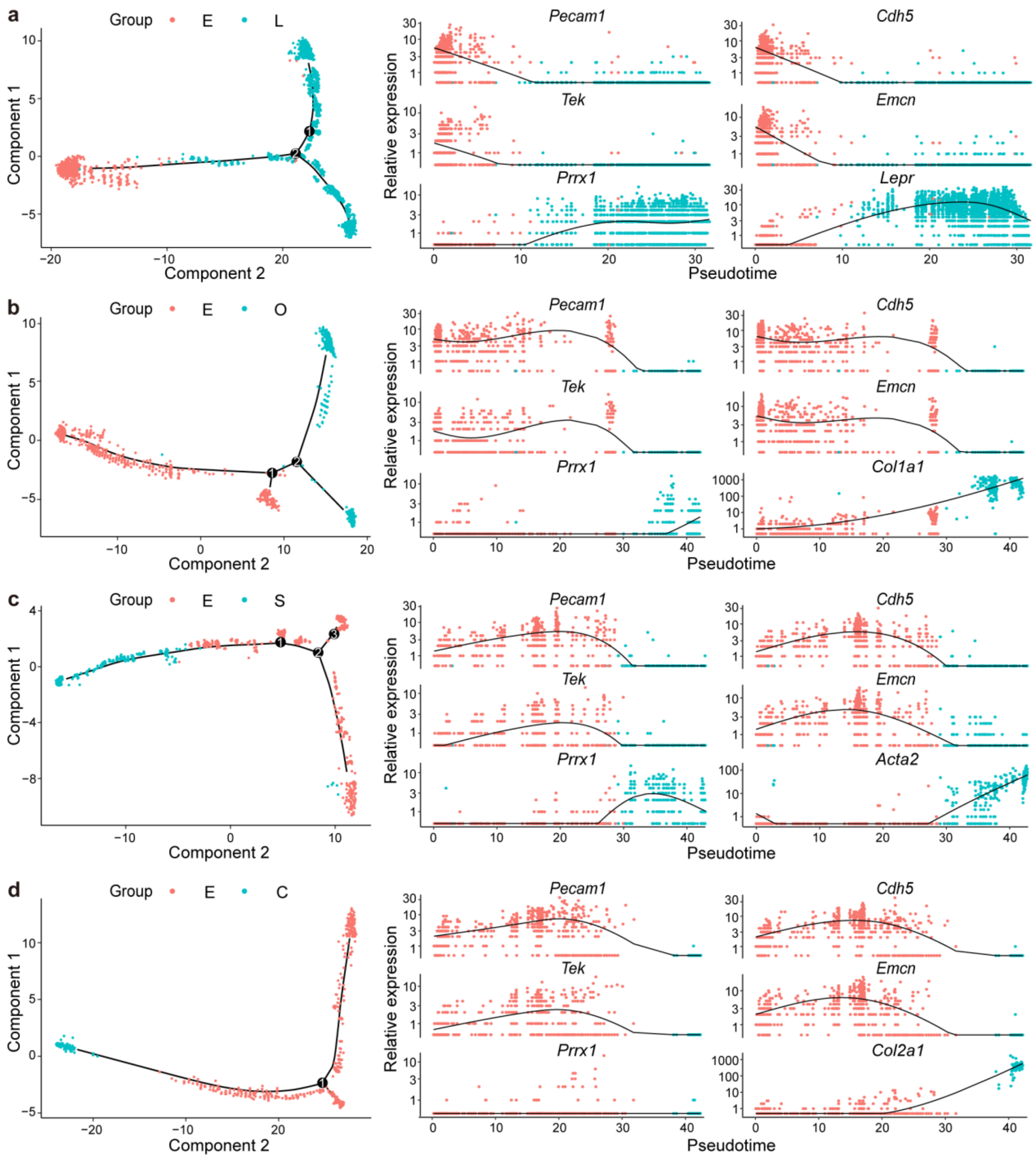

Supplementary Fig. 1 Intermediate cell states between ECs and BMSCs are identified in scRNA-seq analysis. a–d. Pseudotime trajectory analysis between ECs and LEPR^{+} BMSC (**a**), ECs and osteolineage cells (**b**), ECs and SMCs/PCs (**c**), and ECs and chondrolineage cells (**d**) in the scRNA-seq of postnatal mouse bone marrow showing a subset of cells with a gradual decrease in endothelial markers and an increase in mesenchymal markers along the trajectories.

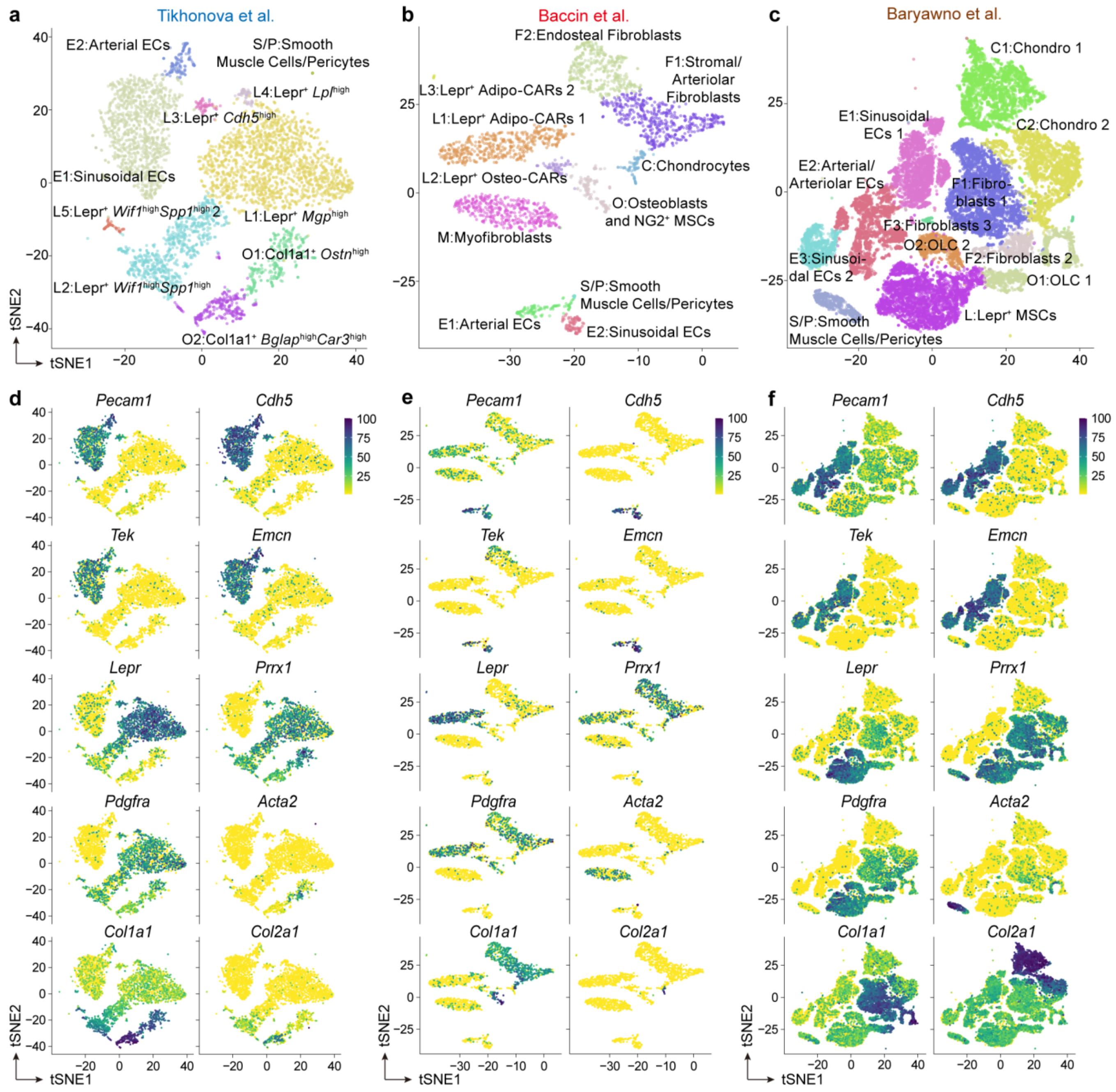

Supplementary Fig. 2 BMSCs expressing endothelial markers are identified in reanalyzed scRNA-seq datasets. a–f. t-SNE visualizations showing clusters of ECs and stromal cells (upper panels) and their expression of endothelial and mesenchymal markers (lower panels) in publicly available scRNA-seq datasets that characterized BMSCs: from lineage tracing models (Tikhonova et al.[19]) (**a**, **d**, n = 4,499 ECs and BMSCs, mice aged between 5–22 weeks); combining spatial transcriptomics (Baccin et al.[22]) (**b**, **e**, n

= 2,453 ECs and BMSCs, mice aged between 8–12 weeks); with several replicates (Baryawno et al.[23]) (**c**, **f**, n = 25,078 ECs and BMSCs, mice aged between 8–10 weeks). E, ECs. L, LEPR$^+$ cells. O, osteolineage cells. S/P, SMCs/PCs. C, chondrolineage cells. F, fibroblasts.

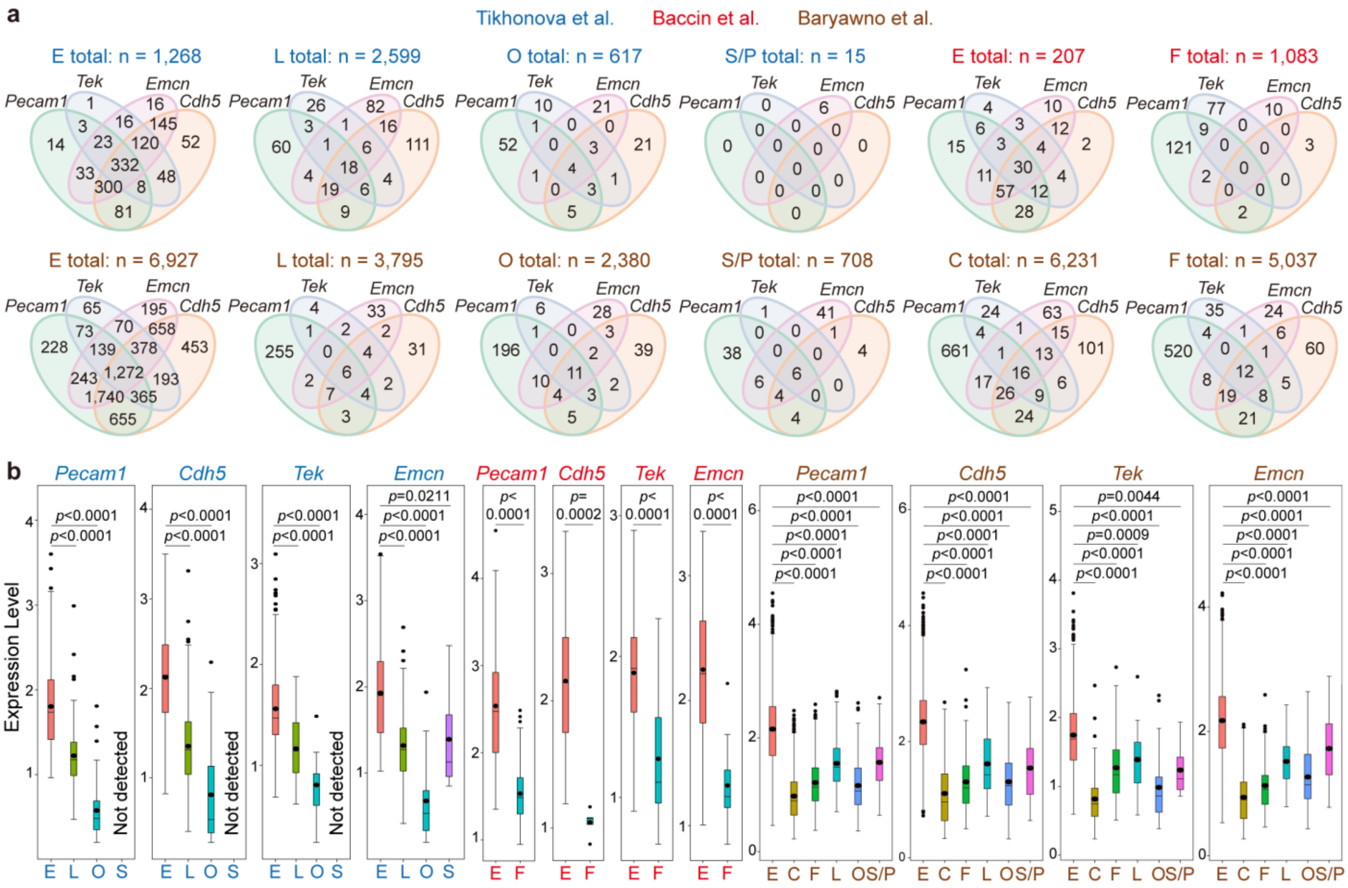

Supplementary Fig. 3 BMSCs primarily present single endothelial markers at low levels in reanalyzed scRNA-seq datasets. a–b. The co-expression (**a**) and the transcript levels of endothelial markers (**b**) in EC and stromal cells expressing these endothelial markers in the reanalyzed scRNA-seq datasets. Statistical significance was determined by two-tailed Wilcox rank-sum test. Source data are provided as a Source Data file.

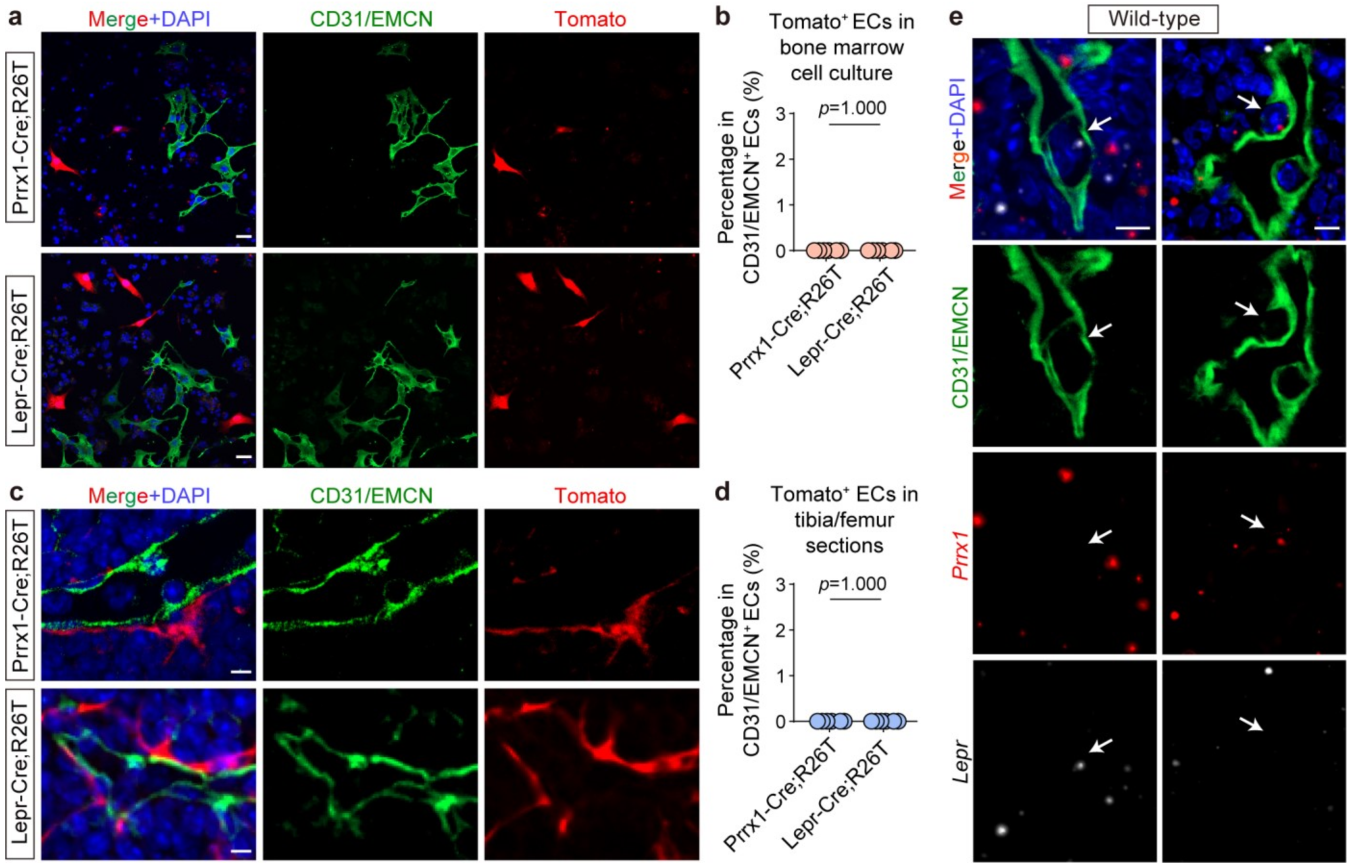

Supplementary Fig. 4 Bone marrow ECs are not labeled by *Prrx1-Cre* and *Lepr-Cre* transgenic mouse models. a. Immunostaining analysis of EGM-cultured bone marrow cells (**a**, **b**) and tibia/femur sections (**c**, **d**) revealing the absence of Tomato^{+} cells in CD31/EMCN^{+} ECs from Prrx1-Cre;R26T (n = 6 biologically independent animals) and Lepr-Cre;R26T (n = 6 biologically independent animals) mice. **e**. RNAscope ISH analysis of tibia/femur sections from wild-type mice (n = 3 biologically independent animals) demonstrating the presence of *Prrx1* and the *ObRb* splice of LEPR in a small number of CD31/EMCN^{+} ECs.

Scale bars: 5 µm. Data represent the mean ± S.E.M.. Statistical significance was determined by two-tailed unpaired Student's *t*-test. Source data are provided as a Source Data file.

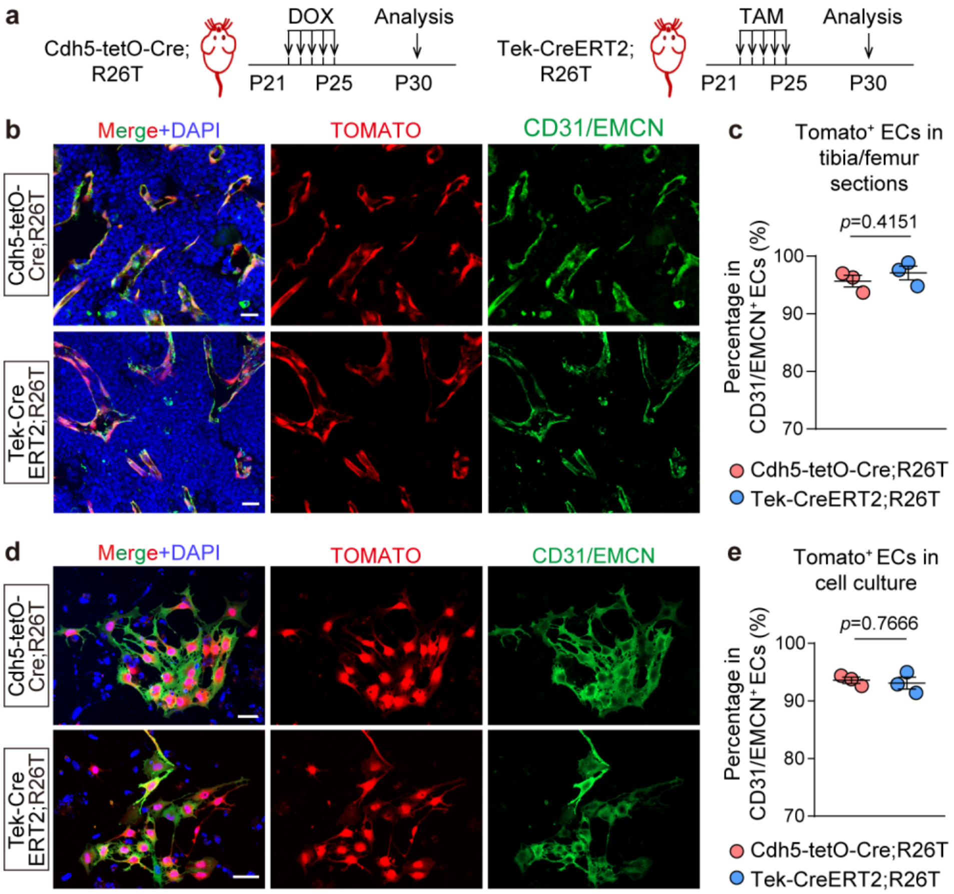

Supplementary Fig. 5 EC lineage tracing models effectively label bone marrow ECs. **a**. Diagrams of protocols. **b**–**e**. Immunostaining analysis of the tibia/femur sections (**b**, **c**) and EGM-cultured bone marrow cells (**d**, **e**) from Cdh5-tetO-Cre;R26T (n = 3 biologically independent animals) and Tek-CreERT2-R26T (n = 3 biologically independent animals) mice showing the effectiveness of these models in labeling bone marrow CD31/EMCN^{+} ECs.

Scale bars: 10 µm. Data represent the mean ± S.E.M.. Statistical significance was determined by two-tailed unpaired Student's t-test. Source data are provided as a Source Data file.

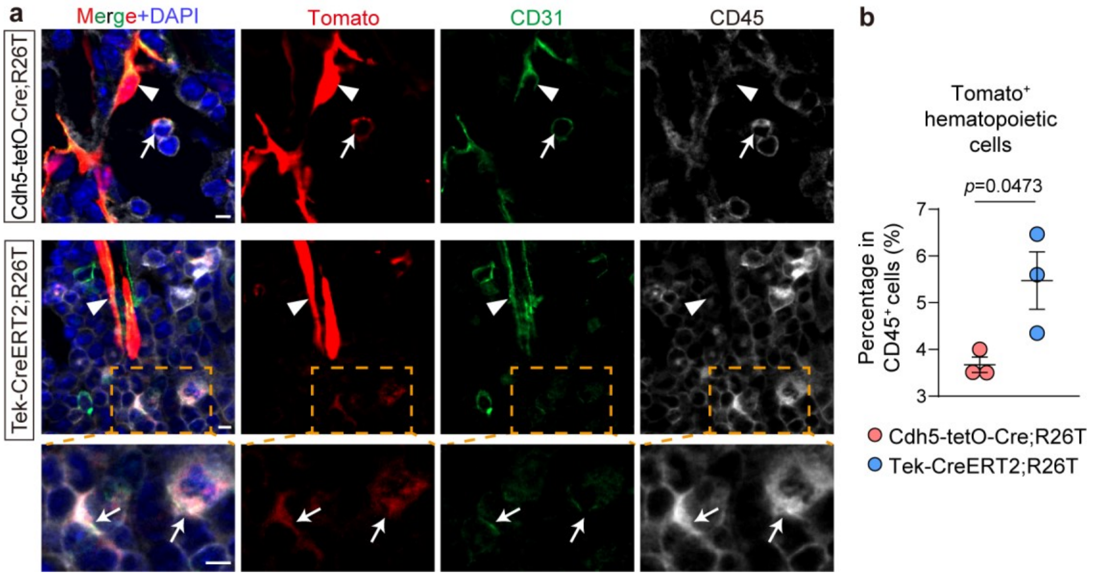

Supplementary Fig. 6 EC lineage tracing models label small subsets of bone marrow hematopoietic cells. **a**–**b**. Immunostaining analysis showing the presence of Tomato^{+} hematopoietic cells (arrows) and ECs (arrowheads) in tibia/femur sections from Cdh5-tetO-Cre;R26T (*n* = 3 biologically independent animals) and Tek-CreERT2-R26T (*n* = 3 biologically independent animals) mice.

Scale bars: 5 µm. Data represent the mean ± S.E.M.. Statistical significance was determined by two-tailed unpaired Student's *t*-test. Source data are provided as a Source Data file.

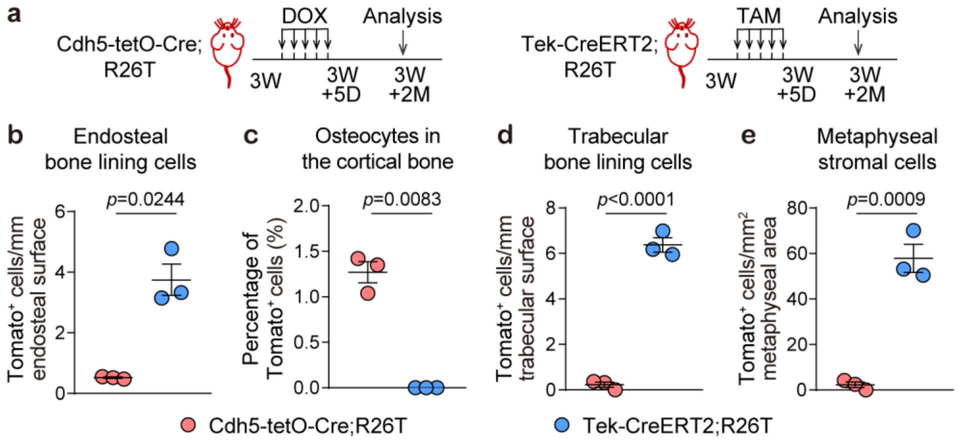

Supplementary Fig. 7 Spatial distribution of endothelial marker-expressing BMSCs does not change in a two-month chase after doxycycline/tamoxifen administration. **a**. Diagrams of protocols. Notes on the timeline indicate the age of mice. **b**–**e**. Quantification of Tomato$^+$ endosteal bone lining cells (**b**), osteocytes in the cortical bone (**c**), trabecular bone lining cells (**d**), and metaphyseal stromal cells (**e**) traced in the Cdh5-tetO-Cre;R26T (n = 3 biologically independent animals) and Tek-CreERT2-R26T (n = 3 biologically independent animals) models.

Data represent the mean ± S.E.M.. Statistical significance was determined by two-tailed unpaired Student's *t*-test (**b**–**c** adjusted for unequal variances with Welch's test). Source data are provided as a Source Data file.

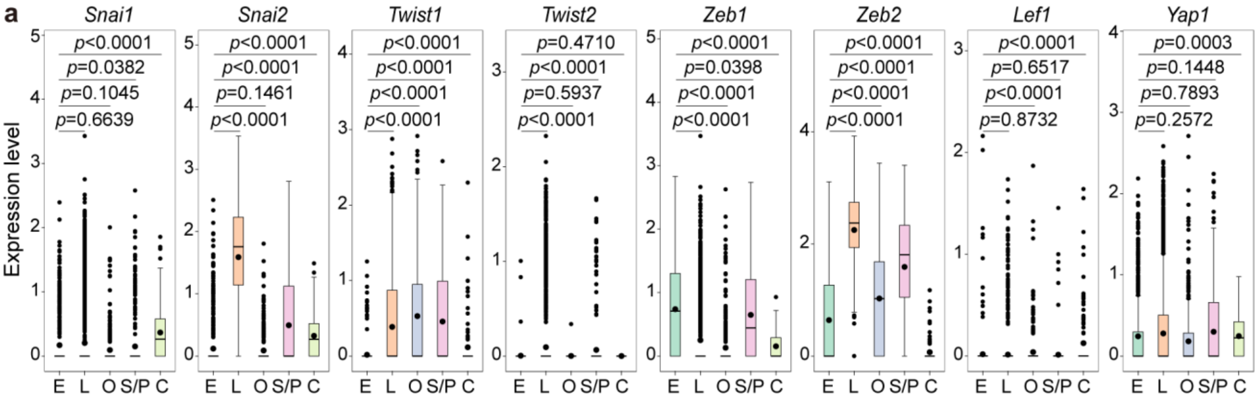

Supplementary Fig. 8 EndoMT-related genes are upregulated in BMSCs than ECs. a. Boxplots showing the expression of EndoMT-related genes among ECs and stromal cell subtypes in the scRNA-seq analysis of collagenase-digested bone and bone marrow cells from 5-week-old wild-type mice. Statistical significance was determined by two-tailed Wilcox rank-sum test. Source data are provided as a Source Data file.

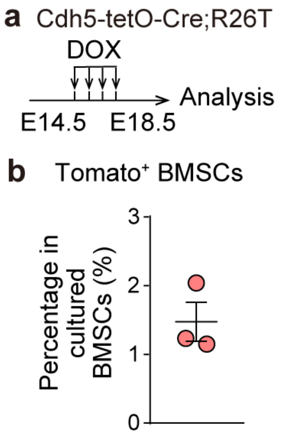

Supplementary Fig. 9 EC lineage tracing model labels small subsets of BMSCs during embryonic development. **a**. Diagrams of protocols. **b**. Flow cytometry analysis showing the percentage of Tomato^{+} cells within cultured BMSCs from E18.5 Cdh5-tetO-Cre;R26T (*n* = 3 biologically independent samples) mice administered with doxycycline from E14.5 to E18.5.

E, embryonic. Data represent the mean ± S.E.M.. Source data are provided as a Source Data file.
