# Supplementary material for: Reassessing endothelial-to-mesenchymal transition in mouse bone marrow: insights from lineage tracing models
Source: Nat Commun. 2023 Dec 20;14:8461. doi: 10.1038/s41467-023-44312-w (PMC10733381; doi:10.1038/s41467-023-44312-w)
Supplement: Supplementary file 2 — Description of Additional Supplementary Files [file 41467_2023_44312_MOESM2_ESM.pdf]

Description of Additional Supplementary Files

File name: Supplementary Data 1

Description: Cluster annotation in the scRNA-seq of postnatal mouse bone marrow cells.

File name: Supplementary Data 2

Description: Cluster identification in the reanalyzed scRNA-seq datasets.

File name: Supplementary Data 3

Description: Identification of EC subclusters in the scRNA-seq of postnatal mouse bone marrow cells.
