# Supplementary material for: Reassessing endothelial-to-mesenchymal transition in mouse bone marrow: insights from lineage tracing models
Source: Nat Commun. 2023 Dec 20;14:8461. doi: 10.1038/s41467-023-44312-w (PMC10733381; doi:10.1038/s41467-023-44312-w)
Supplement: Supplementary file 6 — Reporting Summary [file 41467_2023_44312_MOESM6_ESM.pdf]

nature portfolio

Corresponding author(s): Hui Xie, Zhen-Xing Wang

Last updated by author(s): Nov 23, 2023

Reporting Summary

Nature Portfolio wishes to improve the reproducibility of the work that we publish. This form provides structure for consistency and transparency in reporting. For further information on Nature Portfolio policies, see our Editorial Policies and the Editorial Policy Checklist.

Statistics

For all statistical analyses, confirm that the following items are present in the figure legend, table legend, main text, or Methods section.

n/a	Confirmed	
☐	☒	The exact sample size (*n*) for each experimental group/condition, given as a discrete number and unit of measurement
☐	☒	A statement on whether measurements were taken from distinct samples or whether the same sample was measured repeatedly
☐	☒	The statistical test(s) used AND whether they are one- or two-sided<br>*Only common tests should be described solely by name; describe more complex techniques in the Methods section.*
☒	☐	A description of all covariates tested
☐	☒	A description of any assumptions or corrections, such as tests of normality and adjustment for multiple comparisons
☐	☒	A full description of the statistical parameters including central tendency (e.g. means) or other basic estimates (e.g. regression coefficient) AND variation (e.g. standard deviation) or associated estimates of uncertainty (e.g. confidence intervals)
☐	☒	For null hypothesis testing, the test statistic (e.g. *F*, *t*, *r*) with confidence intervals, effect sizes, degrees of freedom and *P* value noted<br>*Give P values as exact values whenever suitable.*
☒	☐	For Bayesian analysis, information on the choice of priors and Markov chain Monte Carlo settings
☒	☐	For hierarchical and complex designs, identification of the appropriate level for tests and full reporting of outcomes
☐	☒	Estimates of effect sizes (e.g. Cohen's *d*, Pearson's *r*), indicating how they were calculated

Our web collection on statistics for biologists contains articles on many of the points above.

Software and code

Policy information about availability of computer code

Data collection	Illumina NovaSeq 6000, Zen blue (2.3), Flowjo CE, FACS Diva (6.1.3).
Data analysis	ScRNA-seq: 10x Genomics Cell Ranger (4.0.0) and Seurat 3.0. Flow cytometry analysis: FlowJo (V10). Statistical tests: GraphPad Prism (v8.4) and R (4.0.2). Immunofluorescence analysis: Zen blue (2.3), Image J (1.54f).

For manuscripts utilizing custom algorithms or software that are central to the research but not yet described in published literature, software must be made available to editors and reviewers. We strongly encourage code deposition in a community repository (e.g. GitHub). See the Nature Portfolio guidelines for submitting code & software for further information.

Data

Policy information about availability of data

All manuscripts must include a data availability statement. This statement should provide the following information, where applicable:

- Accession codes, unique identifiers, or web links for publicly available datasets
- A description of any restrictions on data availability
- For clinical datasets or third party data, please ensure that the statement adheres to our policy

The raw scRNAseq data are deposited in the NCBI GEO under accession code GSE168333 (https://www.ncbi.nlm.nih.gov/geo/query/acc.cgi?acc=GSE168333). The reanalyzed datasets were obtained from the GEO database under the following accession codes: GSM2915578, GSM2915579, GSE122465, GSM3674224,

GSM3674225, GSM3674226, GSM3674227, GSM3674228, and GSM3674229. All other data are available within the article, Supplementary Information file, Source data file, or from the corresponding authors upon reasonable request. Source data are provided with this paper.

Research involving human participants, their data, or biological material

Policy information about studies with human participants or human data. See also policy information about sex, gender (identity/presentation), and sexual orientation and race, ethnicity and racism.

Reporting on sex and gender	*Use the terms sex (biological attribute) and gender (shaped by social and cultural circumstances) carefully in order to avoid confusing both terms. Indicate if findings apply to only one sex or gender; describe whether sex and gender were considered in study design; whether sex and/or gender was determined based on self-reporting or assigned and methods used.*<br>*Provide in the source data disaggregated sex and gender data, where this information has been collected, and if consent has been obtained for sharing of individual-level data; provide overall numbers in this Reporting Summary. Please state if this information has not been collected.*<br>*Report sex- and gender-based analyses where performed, justify reasons for lack of sex- and gender-based analysis.*
Reporting on race, ethnicity, or other socially relevant groupings	*Please specify the socially constructed or socially relevant categorization variable(s) used in your manuscript and explain why they were used. Please note that such variables should not be used as proxies for other socially constructed/relevant variables (for example, race or ethnicity should not be used as a proxy for socioeconomic status).*<br>*Provide clear definitions of the relevant terms used, how they were provided (by the participants/respondents, the researchers, or third parties), and the method(s) used to classify people into the different categories (e.g. self-report, census or administrative data, social media data, etc.)*<br>*Please provide details about how you controlled for confounding variables in your analyses.*
Population characteristics	*Describe the covariate-relevant population characteristics of the human research participants (e.g. age, genotypic information, past and current diagnosis and treatment categories). If you filled out the behavioural & social sciences study design questions and have nothing to add here, write "See above."*
Recruitment	*Describe how participants were recruited. Outline any potential self-selection bias or other biases that may be present and how these are likely to impact results.*
Ethics oversight	*Identify the organization(s) that approved the study protocol.*

Note that full information on the approval of the study protocol must also be provided in the manuscript.

Field-specific reporting

Please select the one below that is the best fit for your research. If you are not sure, read the appropriate sections before making your selection.

☒ Life sciences ☐ Behavioural & social sciences ☐ Ecological, evolutionary & environmental sciences

For a reference copy of the document with all sections, see nature.com/documents/nr-reporting-summary-flat.pdf

Life sciences study design

All studies must disclose on these points even when the disclosure is negative.

Sample size	No statistical method was used to predetermine the sample size. Required experimental sample sizes were estimated based on previous established protocols in the field. All sample sizes are listed in detail in the figure legends and methods section.
Data exclusions	In the scRNA-seq analysis of the bo/bm samples and the publicly available datasets, the following cells were excluded from analysis: (1) Cells with more than 20% mitochondrial gene expression. (2) Cells in the top 2% quantile of nGene and nUMI. (3) Cells with the value of log10(GenesPerUMI) no more than 0.8. (4) Hematopoietic clusters and small clusters without clear characteristics of BMSCs or ECs.
Replication	Data for the biological replicates were shown in the figures.<br>No replication was attempted for scRNA-seq. In our experimental design, based on the specific objectives and constraints of our research, we did not perform replication for scRNA-seq. Furthermore, to strengthen and validate our findings, we supplemented our primary data with a reanalysis of three different, previously published scRNA-seq studies. The incorporated data from these additional sources effectively compensate for the limited sample size in our primary experiment.
Randomization	No randomization techniques was used. Mice were allocated to experiments randomly and samples were processed in an arbitrary order.
Blinding	The investigators were not blinded to experiment allocation or outcome assessment. Dimension reduction and clustering of the scRNA-seq data were performed using computational tools in a unbiased fashion.

Reporting for specific materials, systems and methods

We require information from authors about some types of materials, experimental systems and methods used in many studies. Here, indicate whether each material, system or method listed is relevant to your study. If you are not sure if a list item applies to your research, read the appropriate section before selecting a response.

Materials & experimental systems

n/a	Involved in the study	
☐	☒	Antibodies
☒	☐	Eukaryotic cell lines
☒	☐	Palaeontology and archaeology
☐	☒	Animals and other organisms
☒	☐	Clinical data
☒	☐	Dual use research of concern
☒	☐	Plants

Methods

n/a	Involved in the study	
☒	☐	ChIP-seq
☐	☒	Flow cytometry
☒	☐	MRI-based neuroimaging

Antibodies

Antibodies used

Antibodies used in flow cytometry:
CD16/CD32 (Biolegend, clone: 93, cat#: 101320, 1:200),
CD31 (Biolegend, clone: 390, cat#: 303136, 1:100),
Emcn (eBioscience, clone: V.7C7, cat#: 50-5851-82, 12-5851-82, and 53-5851-82, 1:100),
TIE2 (eBioscience, clone: TEK4, cat#: 17-5987-82, 1:100),
CDH5 (BD OptiBuild, clone: 11D4.1, cat#: 747749, 1:100),
PDGFRα (BD Pharmingen, clone: APA5, cat#: 562777, and 562774, 1:100),
CD51 (Elabscience, clone: RMV-7, cat#: E-AB-F1235E, 1:100),
Sca-1 (Biolegend, clone D7, cat#: 108111, 1:100),
CD44 (Biolegend, clone IM7, cat#: 103011, 1:100),
CD29 (Biolegend, clone HMβ1-1, cat#: 102215, 1:100),
CD45 (BD Pharmingen, clone: 30-F11, cat#: 550994, and 557659, 1:100),
Lineage cocktail (BD Pharmingen, including Ly-76, Ly-6G/Ly-6C, B220, CD11b, CD3e, clones TER119, RB6-8C5, RA3-6B2, M1/70, 145-2C11, respectively, cat#: 561317, 1:50),
CD71 (BD Pharmingen, clone: C2, cat#: 562858, 1:100),
CD3 (Cell Signaling Technology, clone: 17A2, cat#: 24265s, 1:200),
CD19 (eBioscience, clone: 1D3, cat#: 17-0193-80, 1:200),
Ly6G (BD Pharmingen, clone: 1A8, cat#: 560599, 1:100),
CD11b (BD Pharmingen, clone: M1/70, cat#: 557396, 1:100),
BV711 rat IgG2a κ isotype control (Biolegend, clone: RTK2758 cat#: 400551, 1:100),
BV421 rat IgG2a κ isotype control (BD Horizon, clone: R35-95 cat#: 562602, 1:100),
eFluor660 rat IgG2a κ isotype control (eBioscience, clone: eBR2a cat#: 50-4321-82, 1:100),
APC rat IgG1 κ isotype control (eBioscience, clone: eBRG1, cat#: 17-4301-82, 1:100),
Zombie Aqua dye (Biolegend, cat#: 423101, 1:1000).

Primary antibodies for immunostainings:
CD31 (Abcam, cat#: ab28364, 1:50 or R&D Systems, cat#: FAB3628G, 1:100),
Endomucin (Santa Cruz, cat#: sc-65495, 1:200),
Alpha smooth muscle actin (Abcam, cat#: ab124964, 1:400),
CD45(BD Pharmingen, cat#: 557659, 1:200),
RUNX2(Cell Signaling Technology, cat#: 12556S, 1:400).

Secondary antibodies for immunostainings (all from Jackson ImmunoResearch):
Donkey anti-rabbit Alexa Fluor 488 (711-545-152, 1:400) and Alexa Fluor 647 (711-605-152, 1:400).
Donkey anti-rat Alexa Fluor 488 (712-545-150, 1:400), Alexa Fluor 594 (712-585-150, 1:400) and Alexa Fluor 647 (712-605-150, 1:400).
Donkey anti-goat Alexa Fluor 488 (705-545-147, 1:400).

RNA-ISH probes:
Prrx1 (ACD 485231-C2, hybridizing NM_011127.2, nt 254–1,726),
Lepr (ACD 471171, hybridizing NM_146146.2, nt 3220–4109).

Validation

The antibodies have been validated by the companies and users, as indicated in the product information and citations of these antibodies on the manufacturer's website.

Animals and other research organisms

Policy information about studies involving animals; ARRIVE guidelines recommended for reporting animal research, and Sex and Gender in Research

Laboratory animals

The following transgenic mouse strains were used:
C57BL/6, B6.Cg-Gt(ROSA)26Sortm9(CAG-tdTomato)Hze/J (The Jackson Laboratory), B6.Cg-Tg(Prrx1-cre)1Cjt/J (The Jackson Laboratory), B6.129(Cg)-Leprtm2(cre)Rck/J (The Jackson Laboratory), Tg(Tek-cre/ERT2)1Arnd (EMMA), B6;129S-Cdh5tm1(rtTA-tetO-Cre)Smoc (Shanghai Model Organisms Center NM-KI-18006).

Wild animals

The study did not involve wild animals.

Reporting on sex	Pooled sample from both male and female mice were used in the FACS experiments and male mice were used in other experiments at the indicated postnatal ages. To generate Prrx1-Cre;R26T and Tek-CreERT2;R26T double transgenic mice, male Prrx1-Cre or Tek-creERT2 mice were bred with female Rosa26-LSL-tdTomato mice. For doxycycline treatment, juvenile mice and pregnant dams were orally administered with doxycycline (MCE, HY-N0565B) in their drinking water.
Field-collected samples	The study did not involve field-collected samples.
Ethics oversight	All experiments were performed according to the ethics approvals of the Institutional Animal Care and Use Committee of Central South University, Changsha, Hunan, China (Approval#: 2020sydw0800). Mice were maintained at a maximum of five mice per cage under a standard 12 h light/dark cycle, and had access to food and water ad libitum.

Note that full information on the approval of the study protocol must also be provided in the manuscript.

Plants

Seed stocks	*Report on the source of all seed stocks or other plant material used. If applicable, state the seed stock centre and catalogue number. If plant specimens were collected from the field, describe the collection location, date and sampling procedures.*
Novel plant genotypes	*Describe the methods by which all novel plant genotypes were produced. This includes those generated by transgenic approaches, gene editing, chemical/radiation-based mutagenesis and hybridization. For transgenic lines, describe the transformation method, the number of independent lines analyzed and the generation upon which experiments were performed. For gene-edited lines, describe the editor used, the endogenous sequence targeted for editing, the targeting guide RNA sequence (if applicable) and how the editor was applied.*
Authentication	*Describe any authentication procedures for each seed stock used or novel genotype generated. Describe any experiments used to assess the effect of a mutation and, where applicable, how potential secondary effects (e.g. second site T-DNA insertions, mosiacism, off-target gene editing) were examined.*

Flow Cytometry

Plots

Confirm that:

☒ The axis labels state the marker and fluorochrome used (e.g. CD4-FITC).

☒ The axis scales are clearly visible. Include numbers along axes only for bottom left plot of group (a 'group' is an analysis of identical markers).

☒ All plots are contour plots with outliers or pseudocolor plots.

☒ A numerical value for number of cells or percentage (with statistics) is provided.

Methodology

Sample preparation	Sample preparation for uncultured bone marrow cells<br>Tibiae and femurs were collected and cleaned as described above and cut into small pieces in the digestion medium. The samples were then incubated in an orbital shaker at 120 r.p.m., 37 °C for 45 minutes, filtered through cell strainers, and centrifuged. The pellet was resuspended and subjected to flow cytometry analysis. Alternatively, the resuspended cells were depleted for lineage-positive cells using a lineage cell depletion kit (MiltenyiBiotec, 130-090-858) as per the manufacturer's instructions before flow cytometry analysis.<br><br>Sample preparation for cultured bone marrow cells<br>The culture medium was aspirated, and the cells were washed with PBS. Subsequently, accutase was added to the culture vessels to detach the cells while preserving their endothelial markers. The detached cells were then collected and used for subsequent analyses.
Instrument	FACSCanto II Cell Analyzer (BD Biosciences), Aurora Analyzer (Cytek Biosciences), FACSAria II Cell Sorter (BD Biosciences).
Software	FlowJo (Tree Star) software.
Cell population abundance	Indicated in respective figures.
Gating strategy	To identify singlet cells, a gate was drawn based on the Forward Scatter Area (FSC-A) versus width (FSC-W) or height (FSC-H) features, and dead cells were distinguished using Zombie Aqua dye (Biolegend, 423101, 1:1000).

☒ Tick this box to confirm that a figure exemplifying the gating strategy is provided in the Supplementary Information.
